# Physiologically Based Pharmacokinetic/Pharmacodynamic Modeling to Predict the Impact of CYP2C9 Genetic Polymorphisms, Co-Medication and Formulation on the Pharmacokinetics and Pharmacodynamics of Flurbiprofen

**DOI:** 10.3390/pharmaceutics12111049

**Published:** 2020-11-02

**Authors:** Ioannis Loisios-Konstantinidis, Rodrigo Cristofoletti, Masoud Jamei, David Turner, Jennifer Dressman

**Affiliations:** 1 Institute of Pharmaceutical Technology, Goethe University, Max-von-Laue str. 9, 60438 Frankfurt am Main, Germany; loisios-konstantinidis@em.uni-frankfurt.de; 2 Center for Pharmacometrics and Systems Pharmacology, Department of Pharmaceutics, College of Pharmacy, University of Florida, Orlando, FL 32827, USA; rcristofoletti@cop.ufl.edu; 3 Certara UK Limited, Simcyp Division, 1 Concourse Way, Sheffield S1 2BJ, UK; masoud.jamei@certara.com (M.J.); david.turner@certara.com (D.T.); 4 Fraunhofer Institute of Translational Pharmacology and Medicine (ITMP), Carl-von-Noorden Platz 9, 60596 Frankfurt am Main, Germany

**Keywords:** physiologically based pharmacokinetic (PBPK) modeling, pharmacokinetics/pharmacodynamics (PK/PD), *in vitro in vivo* extrapolation (IVIVE), mechanistic oral absorption modeling, drug–drug interaction (DDI), pharmacogenetics

## Abstract

Physiologically based pharmacokinetic/pharmacodynamic (PBPK/PD) models can serve as a powerful framework for predicting the influence as well as the interaction of formulation, genetic polymorphism and co-medication on the pharmacokinetics and pharmacodynamics of drug substances. In this study, flurbiprofen, a potent non-steroid anti-inflammatory drug, was chosen as a model drug. Flurbiprofen has absolute bioavailability of ~95% and linear pharmacokinetics in the dose range of 50–300 mg. Its absorption is considered variable and complex, often associated with double peak phenomena, and its pharmacokinetics are characterized by high inter-subject variability, mainly due to its metabolism by the polymorphic CYP2C9 (fmCYP2C9 ≥ 0.71). In this study, by leveraging *in vitro, in silico* and *in vivo* data, an integrated PBPK/PD model with mechanistic absorption was developed and evaluated against clinical data from PK, PD, drug-drug and gene-drug interaction studies. The PBPK model successfully predicted (within 2-fold) 36 out of 38 observed concentration-time profiles of flurbiprofen as well as the CYP2C9 genetic effects after administration of different intravenous and oral dosage forms over a dose range of 40–300 mg in both Caucasian and Chinese healthy volunteers. All model predictions for C_max_, AUC_inf_ and CL/F were within two-fold of their respective mean or geometric mean values, while 90% of the predictions of C_max_, 81% of the predictions of AUC_inf_ and 74% of the predictions of Cl/F were within 1.25 fold. In addition, the drug-drug and drug-gene interactions were predicted within 1.5-fold of the observed interaction ratios (AUC, C_max_ ratios). The validated PBPK model was further expanded by linking it to an inhibitory *E_max_* model describing the analgesic efficacy of flurbiprofen and applying it to explore the effect of formulation and genetic polymorphisms on the onset and duration of pain relief. This comprehensive PBPK/PD analysis, along with a detailed translational biopharmaceutic framework including appropriately designed biorelevant *in vitro* experiments and *in vitro-in vivo* extrapolation, provided mechanistic insight on the impact of formulation and genetic variations, two major determinants of the population variability, on the PK/PD of flurbiprofen. Clinically relevant specifications and potential dose adjustments were also proposed. Overall, the present work highlights the value of a translational PBPK/PD approach, tailored to target populations and genotypes, as an approach towards achieving personalized medicine.

## 1. Introduction

Intrinsic and extrinsic patient factors (IEFs) such as dosage form, co-medication, and genetic polymorphism may significantly impact drug exposure and subsequently lead to changes in the efficacy or safety of a drug. The ability to quantify such factors on the exposure and pharmacologic action of a drug would represent a milestone in determining required dose adjustments and implementation of risk management strategies. Under the prism of model-informed drug discovery and development (MID3), dynamic mechanistic models such as whole body physiologically based pharmacokinetic/pharmacodynamic (PBPK/PD) models may be useful for forecasting the influence as well as the interaction of multiple factors on pharmacokinetics (PK) and pharmacodynamics (PD), and as a result could be used to guide formulation selection and clinical dosing recommendations.

Flurbiprofen (FLU) is a potent non-steroid anti-inflammatory drug (NSAID) that has been used as the racemate for the symptomatic treatment of rheumatoid arthritis and osteoarthritis. FLU is a typical acidic representative of class II of the Biopharmaceutics Classification System (BCS), exhibiting very poor solubility in gastric conditions, but high solubility and permeability in the small intestine. FLU is entirely absorbed from the small intestine with a fraction absorbed (*f_a_*) typically greater than 95%, while its absolute bioavailability ranges between 92% and 96% [1]. Even though it is almost completely absorbed, the intestinal absorption of FLU is considered complex and variable, since it is often associated with double peak phenomena and high inter-individual variability in plasma concentrations (up to 80–100%) [1,2,3]. The clinical PK of FLU is stereo-selective, with only the S-enantiomer being pharmacological active, and is linear in the dose range of 50–300 mg. Similar to most NSAIDs, it is highly bound (>99%) to plasma proteins, with a steady-state volume of distribution (V_ss_) of around 0.1 L/kg [1,4,5]. FLU is mainly eliminated by oxidative metabolism in the liver by the cytochrome P450 (CYP) 2C9 to its major metabolite, 4-hydroxy flurbiprofen (4-OH FLU). CYP2C9 metabolic contribution is at least 71% and FLU has been identified as a probe drug for CYP2C9 activity. Further type II biotransformation reactions, such as glucuronidation, are mediated through UGT2B7 and UGT1A9.

CYP2C9 is a polymorphic enzyme, with more than 50 single nucleotide polymorphisms (SNPs) described in the regulatory and coding regions of the CYP2C9 gene. However, of those, only two coding SNPs, namely, CYP2C9*2 and CYP2C9*3, have shown to result in clinically relevant reductions in enzyme activity, while the CYP2C9*1 is the wild type variant [6]. The two afore-mentioned SNPs result in six different genotypes that confer three functionally different phenotypes: (a) extensive metabolizers (EM; CYP2C9*1/*1), (b) intermediate metabolizers (IM; CYP2C9*1/*2, CYP2C9*1/*3, and CYP2C9*2/*2), and poor metabolizers (PM; CYP2C9*2/*3 and CYP2C9*3/*3) [6,7,8]. Although the wild type variant is the most common allele of the CYP2C9 polymorphic family, the frequency of CYP2C9 genetic polymorphisms varies significantly among different ethnic populations [9,10,11]. Thus, increased FLU plasma exposure might be observed in subjects with different genotypes as well as after co-administration of CYP2C9 inhibitors.

PBPK modeling has been increasingly used in recent years for predictions of formulation effects, drug–drug interactions, and pharmacogenetics in drug development and to support regulatory decision-making [12,13,14,15,16,17,18,19,20,21,22]. A translational absorption PBPK/PD modeling approach is required in order to gain mechanistic insight into the effect of multiple intrinsic and extrinsic patient factors on the exposure and therapeutic response of a drug. For that purpose, we generated biorelevant *in vitro* data from multiple FLU formulations, and the biopharmaceutical parameters were then translated to *in vivo* dissolution and absorption scenarios. Leveraging *in vitro, in silico*, and *in vivo* data, we developed a comprehensive integrated PBPK/PD model and evaluated it against clinical PK/PD, pharmacogenetic (PG), and drug–drug interaction studies. In summary, the aim of the present study was to evaluate the impact of formulation, genetic polymorphism, and co-medication on the pharmacokinetics and pharmacodynamics of FLU.

## 2. Materials and Methods

### 2.1. Chemicals and Reagents

FLU (lot #LRAA9230) pure active pharmaceutical ingredient (API) was purchased commercially from Sigma-Aldrich Co., LLC. (St. Louis, MO, USA). Three immediate release (IR) tablet formulations of FLU with qualitatively different compositions were selected for study: (a) 100 mg FLU United States Pharmacopoeia (USP film-coated tablets, lot 3077637; Mylan Pharmaceuticals Inc., Morgantown, WV, USA), (b) 100 mg Antadys (film-coated tablets, lot 8M824; Teva Sante, Paris, France), and (c) 100 mg Froben (sugar-coated tablets, lot 31257J4; BGP Products GmbH, Baar, Switzerland), purchased from the American, French, and Swiss markets, respectively. Fasted state-simulated gastric fluid (FaSSGF), fasted state-simulated intestinal fluid (FaSSIF V1), fed state-simulated intestinal fluid (FeSSIF V1) powder (lot 01-1512-05NP), and FaSSIF V3 powder (lot PHA S 1306023) were kindly donated from Biorelevant.com Ltd. (Surrey, United Kingdom). Acetonitrile (lot 18D181599) and water (lot 17B174006) of HPLC-grade were purchased from VWR Chemicals (Leuven, Belgium). Sodium hydroxide pellets (lot 14A100027), sodium chloride (lot 17I074122), sodium acetate (lot 14B240013), hydrochloric acid 37% (lot 10L060526), orthophosphoric acid 85% (lot 12K210017), and glacial acetic acid 100% (lot 12B220508) were obtained commercially from VWR Chemicals (Leuven, Belgium). Sodium dihydrogen phosphate dehydrate (lot K93701642712) and citric acid (lot K91221207425) were purchased from Merck KGaA (Darmstadt, Germany). Pepsin from porcine gastric mucosa 19.6% was obtained from Sigma-Aldrich Co., LLC.

### 2.2. In Vitro Solubility Experiments

The solubility of FLU was investigated in various aqueous and biorelevant dissolution media using the Uniprep system (Whatman, Piscataway, NJ, USA). All aqueous buffers were prepared according to the European Pharmacopoeia, while the biorelevant media were prepared according to Markopoulos et al. and Fuchs et al. [23,24]. An excess amount of API was added to 3 mL of dissolution medium and the samples were incubated for 24 h at 37 °C on an orbital mixer. The samples were then filtered through the 0.45 μm polytetrafluoroethylene (PTFE) filter integrated in the Uniprep system. The filtrate was immediately diluted with mobile phase and analyzed by high-performance liquid chromatography (HPLC) (see Section 2.5). All measurements were performed at least in triplicate (*n* ≥ 3) and the final pH was recorded.

### 2.3. In Vitro Dissolution Tests

All dissolution tests were performed using a calibrated USP II (paddle) apparatus (Erweka DT 80, Heusenstamm, Germany) at 37 ± 0.4 °C. Each vessel contained 500 mL of fresh, pre-warmed medium and the rotational speed was set at 75 rpm. Samples were withdrawn at 2.5, 5, 10, 15, 20, 30, 45, 60, 90, and 120 min via a 5 mL glass syringe connected to a stainless-steel cannula containing a 10 μm polyethylene cannula filter. Immediately thereafter, the sample was filtered through a 0.45 μm PTFE filter (ReZist 30, GE Healthcare UK Ltd., Buckinghamshire, United Kingdom), discarding the first 2 mL. The filtrate was immediately diluted with mobile phase and analyzed by HPLC-UV (see Section 2.5). The removal of 5 mL at each sampling time was considered in the calculation of the percentage dissolved. All experiments were performed at least in triplicate (*n* ≥ 3) and the final pH in the vessel was recorded.

### 2.4. Two-Stage Dissolution Test

Since the conventional one-stage USP II dissolution test does not include a gastric compartment to account for disintegration of the dosage form in the stomach, differences in the disintegration time between simple film-coated (i.e., 100 mg FLU USP and 100 mg Antadys) and sugar-coated formulations (i.e., 100 mg Froben) might bias the interpretation of the biorelevant *in vitro* dissolution behavior with respect to the *in vivo* performance. Hence, to investigate the disintegration effect on the *in vitro* performance of FLU formulations, we performed a two-stage dissolution test with FaSSIF V3 as the intestinal medium according to Loisios-Konstantinidis et al. [25]

The tested dosage forms were initially exposed to 250 mL of gastric medium (i.e., FaSSGF Levels I and III) and samples were removed at 5, 10, 15, 20, and 30 min and treated as described in Section 2.3. After the withdrawal of the last sample, we added 6.8 mL of sodium hydroxide 1M and immediately thereafter 250 mL of FaSSIF V3 concentrate pH = 6.7 (double concentration of all the constituents, apart from sodium hydroxide) to the vessel. Sodium hydroxide was added first, but almost simultaneously with FaSSIF V3. This was done to avoid using a very high pH in the FaSSIF V3 concentrate. After the pH shift, further samples were removed at 32.5, 35, 40, 45, 50, 60, and 90 min. The two-stage dissolution tests were performed using calibrated USP II (paddle) apparatus (Erweka DT 80, Heusenstamm, Germany) at 37 ± 0.4 °C and the samples were analyzed by HPLC-UV (see Section 2.5). All experiments were performed at least in triplicate (*n* ≥ 3) and the final pH in the vessel was recorded.

### 2.5. Quantitative Analysis of Samples

Samples obtained from solubility and dissolution experiments were first filtered through a 0.45 μm PTFE filter (ReZist 30 syringe filter or Uniprep; Whatman, Piscataway, NJ, USA) and subsequently, after appropriate dilution with mobile phase, analyzed by HPLC-UV (Hitachi Chromaster; Hitachi Ltd., Tokyo, Japan or Spectra System HPLC, ThermoQuest Inc., San Jose, CA, USA). A BDS Hypersil C18, 5 μm, 150 × 4.6 mm (Thermo Scientific, Waltham, MA, United States) analytical column combined with a pre-column (BDS Hypersil C-18, 3μm, 10 × 4mm) was used. The mobile phase consisted of water adjusted to pH = 3.0 with trifluoroacetic acid (TFA) and acetonitrile (49.5:0.5:50% *v*/*v*). The detection wavelength was set at 247 nm, the flow rate at 1.0 mL/min, and the injection volume at 20 μL. Using this method, the retention time was approximately 6.8 min. The limit of detection (LOD) and quantification (LOQ) were 0.03 and 0.05 μg/mL, respectively.

### 2.6. Model-Based Analysis of In Vitro Solubility Data

An experimental estimate of FLU pK_a_ was obtained by fitting the Henderson–Hasselbalch equation (Equation (1)) to the mean aqueous equilibrium solubility $(S_{i})\;$values using the SIVA Toolkit version 3.0 (SIVA 3; Certara, Simcyp Division; Sheffield, UK). The lowest reported value in buffers was assumed to represent the intrinsic solubility $\left(S_{0}\right)$. The pK_a_ was then compared with values available in the literature to confirm the validity of the aqueous solubility parameter estimates.

(1) $$S_{i}=S_{0}\cdot \left(10{\;}^{pH-pKa}\right)$$

The impact of bile salt concentration $\left(\left[BS\right]\right)\;$and subsequent formation of micelles on the solubility of FLU was investigated. This was achieved by mechanistically modelling the mean solubility values in fasted state biorelevant media (*n* = 3), accounting also for the relative proportions of FLU solubilized in the aqueous versus the micellar phases, using the total solubility $\left(S_{\left(BS\right)Tot}\right)$ equation (Equation (2)) in SIVA 3.0. Estimates of the logarithm of the micelle-water partition coefficient for the neutral $\left(K_{m:w,unionized}\right)$ and ionized drug $\left(K_{m:w,ionized}\right)$ were obtained to quantify the micelle-mediated solubility.

(2) $$S_{\left(BS\right)Tot}=\left(\;\left[BS\right]\cdot \frac{S_{0}}{C_{H2O}}\cdot K_{m:w,unionized}+\;S_{0}\right)+\left(\;\left[BS\right]\cdot \frac{S_{i}}{C_{H2O}}\cdot K_{m:w,ionized}+\;S_{i}\right)\;$$

Estimation of the relevant parameters was performed using the Nelder–Mead algorithm with weighting by the reciprocal of the predicted values. All estimates based on the *in vitro* solubility data were used as *in silico* input parameters for the development of the physiologically based pharmacokinetic (PBPK) model.

### 2.7. Model-Based Analysis of In Vitro Dissolution Data

Once confidence in the estimation of solubility-related parameters was established, we performed further model-based analysis of the *in vitro* dissolution data obtained from both one and two-stage tests within the serial dilution module of the SIVA Toolkit (SIVA 3.0). The dissolution rate of spherical particles under sink and non-sink conditions within SIVA is described by an extension of the diffusion layer model (DLM) developed by Wang and Flanagan (Equation (3)) [26,27].

(3) $$DR\left(t\right)=-N\cdot S_{DLM}\cdot \frac{D_{eff}}{h_{eff}\left(t\right)}\cdot 4\pi \cdot \alpha \left(t\right)\cdot \left(\alpha \left(t\right)+h_{eff}\left(t\right)\right)\cdot \left(S_{surface}\left(t\right)-C_{bulk}\left(t\right)\right)$$

where $DR\left(t\right)$ is the dissolution rate at time *t*, *N* is the number of particles in a given particle size bin, and $S_{DLM}\;$is a lumped correction scalar without regard to the mechanistic origin of the correction to the DLM. The S_DLM_ estimates obtained with SIVA can be applied to the Simcyp PBPK simulator to reflect differences between media or formulations by simulating the respective *in vivo* dissolution; $D_{eff}$ is the effective diffusion coefficient; $h_{eff}\left(t\right)$ and $\alpha \left(t\right)$ represent the thickness of the hydrodynamic boundary layer and the particle radius at time *t*, respectively;$\;S_{surface}\left(t\right)$ corresponds to the saturation solubility at the particle surface (which may be different to the bulk fluid solubility, as discussed below); and $C_{bulk}\left(t\right)$ is the concentration of dissolved drug in bulk solution at time t.

The $h_{eff}\left(t\right)$ was calculated by the fluid dynamics sub-model, which enables the hydrodynamic conditions to be described according to local conditions and stirring rate. Fluid dynamics-based $h_{eff}\left(t\right)$ is the recommended option for describing the hydrodynamics, as it permits a more rational translation of estimated parameters such as the $S_{DLM}\;$to *in vivo* conditions, in which the hydrodynamics are usually quite different to *in vitro* experiments.

The local pH at the particle surface of ionizable drugs can significantly affect the $S_{surface}$ and consequently the dissolution rate [28,29,30,31,32,33]. Since the *in vitro* dissolution media have a somewhat higher buffer capacity than the intestinal fluids, the self-buffering effect at the solid surface can be underestimated. For this reason, the surface pH was calculated and directly input into SIVA. The calculation of the surface pH is based on the model first proposed by Mooney et al. [29], which assumes that dissolution is the result of both chemical reaction between the conjugate base of the buffer species and the hydrogen cations released from the dissolving drug (in this case FLU) at the liquid–solid interface and the diffusion of the dissolved particles to the bulk. This model is very similar to the quasi-equilibrium model published by Ozturk et al. [31], a derivative of which is implemented in SIVA as the default option for surface pH calculations.

By fitting the DLM model to the observed dissolution data, we obtained $S_{DLM}\;$estimates for each dissolution and two-stage test. In the case of two-stage testing, different $S_{DLM}$ values were obtained for the gastric and intestinal compartments, accounting for the changes in the respective *in vitro* conditions. Under fasted state intestinal conditions, FLU is freely soluble and therefore dissolution is not expected to be solubility limited. In that case, disintegration of the solid dosage form in the intestinal dissolution medium might be the rate-limiting step for the *in vitro* dissolution rate, especially in single dissolution experiments where the dosage form is directly exposed to the intestinal medium without any pre-treatment in a gastric medium. When disintegration was considerably slower than dissolution, and thus had an impact on the overall dissolution rate, the first-order disintegration option was activated in SIVA and used to obtain estimates of a first-order disintegration rate constant $\left(k_{d}\right)$ for those experiments. For the two-stage test experiments, the option was kept deactivated since disintegration in the stomach is already accounted for by the dissolution in the gastric medium. Both gastric and intestinal phases of the two-stage results were modelled simultaneously using the serial dilution model, which can account for more than one *in vitro* dissolution condition in the same experiment.

Estimation of the relevant parameters was performed using the Nelder–Mead algorithm and equal weighting was applied. The various estimated $S_{DLM}$ and $k_{d}$ values were implemented in the Simcyp Simulator (V18.1; Certara, Sheffield, UK) to simulate various *in vivo* dissolution scenarios for the formulations under study and to generate *in vitro–in vivo* extrapolation relationships. These are necessary to predict the *in vivo* performance of the pure drug or formulation using PBPK modelling.

### 2.8. Clinical Studies

#### 2.8.1. PBPK Development and Evaluation Studies

A total of 17 plasma concentration–time profiles from 10 clinical trials published in the open literature were used in support of the development and validation of the FLU physiologically based PBPK/PD model. Data after intravenous administration were obtained from Mei et al. [34]. In this crossover bioequivalence study, 24 healthy male Chinese subjects were administered a single dose (s.d.) of FLU axetil intravenously after an overnight fast. 

Nine studies were performed after oral administration of a single dose of FLU at different dose levels and dosage forms in the fasted state.In the study by Gonzalez-Younes et al. [35], 12 Caucasian healthy, non-smoker males, aged between 25 and 31 years and weighing within 10% of their ideal body weight (BW) for height (BH), were administered 25 mL of oral solution containing 67.9 mg FLU in the fasted state. In a three-way three-treatment randomized crossover study, Szpunar et al. investigated the linearity of the pharmacokinetics of FLU [36]. In this study, 15 healthy subjects with mean (range) age of 29 (18–40) years old, and weight (range) and height (range) of 76.4 (62.3–109) and 177 (168–188) cm, respectively, were administered single oral doses of 100, 200, and 300 mg as immediate release (IR) tablets. Additionally, in a separate treatment, all participants received 40 mL of oral solution containing 100 mg FLU (2.5 mg/mL). In all treatments, all individuals received the medication at 7:00 a.m. with 180 mL water, after an overnight fast. In a pharmacokinetic study by Lee et al., 13 Korean male healthy volunteers, who had fasted overnight, received an oral solution of 40 mg from pre-dissolved Froben tablets [37]. The latter study also explored the effect CYP2C9-specific genotypes, CYP2C9 1*/1* (wild type) and 1*/3*, on the pharmacokinetics of FLU. Similarly, in the study by Lee et al., the differences in metabolism and pharmacokinetics among individuals with the CYP2C9 1*/1*, 1*/2*, and 1*/3* genotypes were investigated. A total of 15 (5 for each genotype), 8 female and 7 male, healthy Caucasian (one Hispanic) volunteers aged between 24 ± 5 years and weighing 79 ± 18 kg were administered a 50 mg FLU tablet after an overnight fast. As well as taking plasma samples, the researchers also collected pooled urine.

Several clinical studies after oral administration of FLU at its highest strength (100 mg) are available in the open literature [4,36,38,39]. In a relative bioavailability study with a crossover design by Jamali et al., 23 healthy Caucasian male subjects with a mean (range) age of 27.2 (18–35) years old received 100 mg Froben or 100 mg Ansaid with 100 mL water after an overnight fast. The mean (range) body weight was 71.8 (52.5–88.5) kg and all individuals were within 20% of their ideal body weight for their height [38]. In the study by Patel et al., 4 Caucasian (50% females) healthy volunteers with mean (SD) age and weight of 26.8 (2.2) years old and 67.8 (4.1) kg, respectively, took part [4]. All subjects had fasted overnight and on the next morning were administered a 100 mg Froben tablet with approximately 150 mL water. In a randomized, double blind, placebo-controlled, crossover study, Suri et al. investigated the pharmacokinetics (PK) and pharmacodynamics (PD) of FLU after oral administration. In this study, 6 healthy subjects were given 100 mg FLU orally as a single tablet with 200 mL water after an overnight fast, on 2 separate occasions [39]. No further demographic and background characteristics were described. The analgesic efficacy was evaluated by 2 independent pharmacodynamic endpoints, including a subjective pain intensity rating and tooth pulp-evoked potentials (TPEP) amplitude, which is more objective.

To investigate the bioequivalence between orally disintegrating and conventional FLU tablets in a randomized-sequence, open-label, 2-period crossover study, Liu et al. administered a single dose of 150 mg (as 3 tablets of 50 mg) FLU of either the orodispersible (test) or the conventional (reference) formulation to 20 healthy, non-smoking Chinese male volunteers [40]. After a 12 h fast, the subjects received the test product without any water intake, whereas 250 mL water were given with the reference product. The enrolled individuals had a mean (SD) age, weight, height, and body mass index (BMI) of 21.4 (2.5) years, 63.2 (5.1) kg, 174.4 (4.2) cm, and 20.8 (1.4) kg/m^2^, respectively.

In all studies, concomitant administration of any other drugs was not permitted for at least 1 week before the study and food was withheld until 2 h post-dose.

All available demographic data from the aforementioned clinical studies were used in simulations of the clinical trials and they are summarized in Table 1.

#### 2.8.2. Drug–Drug Interaction (DDI) Studies

A total of 13 sets of plasma concentration–time profiles of FLU with or without perpetrator co-administration from a total of 6 clinical studies available in the open literature were used for CYP2C9 drug–drug–gene predictions. In an open randomized crossover study, Kumar et al. investigated the impact of CYP2C9 genotype- and dose-dependent inhibition interactions of FLU *in vivo* [43]. From a total of 189 genotyped subjects, 11 CYP2C9 1*/1*, 8 CYP2C9 1*/3*, and 2 CYP2C9 3*/3* healthy subjects received either 50 mg FLU (Mylan Pharmaceuticals Inc., Maharashtra, India) as a tablet alone or 200 mg or 400 mg fluconazole as tablet once daily (q.d.) for 7 days, followed by 50 mg FLU on the 7th day. Subjects were required to fast overnight prior to the study day and FLU was administered 2 h after administration of the last fluconazole dose. In a total of 3 clinical studies investigating the potential of *in vivo* CYP2C9 inhibition by pomegranate, blueberry, cranberry or grape juice, the researchers used FLU as the index substrate and fluconazole as the inhibitor [44,45,46]. Following the same design and administration protocol, the researchers administered fluconazole to healthy volunteers as a 200 mg tablet twice on the afternoon before the day of study and 30 min prior to the administration of a 100 mg FLU tablet on the study day. After FLU administration, venous blood samples were drawn over 12 h. In addition, Zgheib et al. evaluated the effect of study design, i.e., after administration of either a single or 7 once daily doses of 400 mg fluconazole, on the *in vivo* metabolism and pharmacokinetics of FLU [47]. A total of 12 healthy volunteers completed the study. After overnight fast, 50 mg of FLU was administered as a tablet (Ansaid) 2 hours after the last dose of fluconazole. Daali et al. assessed the usefulness of dried blood spots (DBS) to determine the FLU metabolic ratio by comparing plasma concentration with DBS profiles after 3 treatments: (a) administration of a 50 mg FLU tablet alone, (b) 50 mg of FLU together with a single 400 mg dose of fluconazole as the CYP2C9 inhibitor, and (c) 50 mg of FLU with 5 doses (once daily) of 600 mg rifampicin as the CYP2C9 inducer [48]. FLU administration to 10 healthy male subjects took place 2 hours after fluconazole and concomitantly with the last dose of rifampicin; between treatments there was at least a 2-week washout period.

In all studies, no concomitant administration of any other drugs was permitted for at least 1 week before the start of the study; food was withheld until 2 h post-dose and a washout period of at least 1 week was applied.

All available demographic and study design data of the DDI studies are presented in Table 2.

### 2.9. PBPK Model Development and Verification

#### 2.9.1. Software

PBPK modeling and simulations were performed using the Simcyp Population-based Simulator (V18.2; Certara, Sheffield, United Kingdom). The FLU PBPK model was developed by implementing a “middle-out” stepwise sequential modeling strategy, in line with previously published literature and regulatory guidelines [16,49,50,51,52,53]. Briefly, the initial model was developed through integration of physicochemical parameters, *in vitro* data, and/or *in silico* predictors for the absorption, distribution, metabolism, and excretion (ADME) processes. *In vitro* data generated for the purpose of this study were also incorporated after using an *in vitro–in vivo* extrapolation (IVIVE) approach. All input parameters for the FLU PBPK/PD model are summarized in Table 3. Simulations were performed using the virtual North European Caucasian and Chinese healthy volunteer populations of the software.

#### 2.9.2. PBPK/PD Model Development

Physicochemical Characteristics and Blood Binding

FLU has a molecular weight (MW) of 244.3 g/mol and is a poorly soluble (BCS II) monoprotic acid with a pKa of 4.05. The logarithm of the octanol–water partition coefficient is 3.99 [55,60], while the values for the blood/plasma concentration ratio (B:P) and the fraction unbound (f_u_) are 0.55 and 0.01, respectively [5,56,57,58,59].

##### Absorption

The Advanced Dissolution Absorption and Metabolism (ADAM) model was used to mechanistically describe the absorption of FLU. The ADAM model has previously been described in detail by Jamei et al. and Darwich et al. [63,64]. The human effective permeability (P_eff_) was calculated using *in vitro* apparent permeability (P_app_) data in Caco-2 cells for both the compound and positive (Verapamil)/ negative (Atenolol) calibrators [60]. The P_eff_ was predicted to be 4.83 × 10^−4^ cm/s through using a pH of 6.5 on the apical side of the Caco-2 cells and assuming only passive permeation. The diffusion layer model (DLM) with advanced fluid dynamics (AfD) and dynamic (time variant) pH were implemented to simulate the *in vivo* dissolution. Default settings of the software for luminal blood flow, fluid volume, bile salt content, segmental pH, metabolic activity, and small intestinal residence time were applied. The mean gastric emptying time (GET) in the fasted state was set to 0.25 h (matching the built-in “segregated transit time” model value rather than the default value of 0.4 h used in the “global” transit time model), as suggested by human clinical data and several authors [65,66,67,68]. The S_0_ was set to the minimum experimentally measured value, while estimates for the neutral and ionized species K_m:w_ (Equation (2)) were incorporated after modelling of the *in vitro* biorelevant solubility data (Section 2.6). A dissolution-based IVIVE approach, using S_DLM_ estimates from *in vitro* data, was followed to account for formulation or media-related differences when simulating the respective *in vivo* dissolution scenarios (Section 2.7). Further, to investigate the effect of *in vivo* dissolution of multiple formulations and under various conditions on the overall *in vivo* performance, we implemented selected *S_DLM_* estimates to simulate the aforementioned clinical studies at the 100 mg dose level. At other dose levels, the highest gastric (*S_DLM, stomach_)* and intestinal (*S_DLM, SI_)* estimates corresponding to the fastest gastric and intestinal dissolution rates, respectively, were used to minimize the impact of formulation.

##### Distribution

A full PBPK distribution model was used and distribution parameters including organ/tissue partition coefficients (K_p_) and volume of distribution at the steady state (V_ss_) were predicted by the built-in Method 2 (the Rodgers–Rowland method) [69].

##### Metabolism and Excretion

The contributions of CYP2C9 (f_mCYP2C9_ = 0.71) on the overall metabolic clearance (CL) of FLU as well as the renal clearance (CL_renal_ = 0.66) were obtained from Patel et al. [4]. Using the retrograde model for healthy volunteers available within the PBPK software, we calculated additional liver CL to match the reported f_mCYP2C9_. Using human recombinant (rhP450) CYP2C9 expressed in microsomes from the insect cell line Sf21, we found the mean V_max_ and K_m_ values for the 1*/1* (wild type), 2*/2*, and 3*/3* to be 15.79 and 8.756, 10.04 and 10.39, and 8.901 and 23.25, respectively [61]. These allele-specific CYP2C9 *in vitro* kinetic parameters (V_max_, K_m_) were implemented to further inform the model. The metabolic clearance of heterozygotic subjects with CYP2C9 1*/2* and CYP2C9 1*/3* genotypes has been clinically observed to be 0.73 and 0.605 of the wild type (1*/1*) clearance, respectively [42]. For that reason, and in the absence of *in vitro* data, the V_max_ of CYP2C9 1*/1* was scaled down accordingly to account for the decrease in clearance in those genotypes. The K_m_ value was assumed to be the same as for CYP2C9 1*/1*. All presented V_max_ and K_m_ values were already normalized to account for microsomal incubation fraction unbound (f_u,mic_). Since an inter-system extrapolation factor (ISEF) was not available for this particular rhP450 system, we used a literature ISEF value (equal to 0.38) from baculovirus insect cell-expressed CYP2C9 for another NSAID, diclofenac, as an initial estimate [70]. After oral administration of racemic FLU, 8.4 and 7.3% of the dose was excreted into the urine as the acyl glucuronide of (R)- and (S)-FLU, respectively [4], indicating that glucuronidation made some contribution to the metabolic pathway of FLU. The major UGT isoform involved in FLU glucuronidation is UGT2B7, with minor contributions by UGT1A1, UGT1A3, UGT1A9, and UGT2B4 [71,72]. Even though genetic polymorphisms have been reported in UGT family members [73,74], the clinical and functional significance and genotype–phenotype correlation of UGT polymorphisms is an ongoing area of research. In absence of data showing clinical relevance of UGTB7 and UGT1A9 polymorphisms, these were not considered for the development and validation of the present model.

##### Pharmacodynamics

A published inhibitory E_max_ model linked to an effect-compartment was coupled to the PBPK model for FLU [39]. The analgesic efficacy was assessed using 2 endpoints: (a) subjective pain intensity ranking and (b) tooth pulp-evoked potentials (TPEP) amplitude. The percentage change of each endpoint after drug intake was considered as an indicator of pharmacodynamic activity, while the pre-dose value was defined as 100% (initial value).

##### Model Optimization

The volume of distribution and clearance were further optimized by estimating the K_p_ scalar and the ISEF value, respectively, with simultaneous fitting of the model to PK data after 50 mg intravenous and 67.9 mg oral solution administrations (internal datasets).

#### 2.9.3. PBPK/PD Model Validation and Evaluation of Predictive Performance

**Figure 1:**
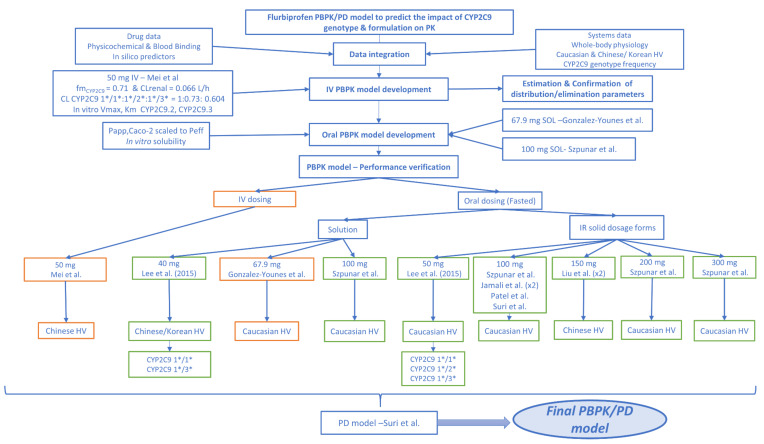
Stepwise modeling workflow for the development and verification of flurbiprofen PBPK/PD model. Training for the internal and test datasets for the external verification, obtained from clinical studies published in the open literature, are outlined with orange and green, respectively.

The performance of the developed PBPK/PD model was evaluated by clinical trial simulations. In order to assess the distribution of population variability, we simulated at least 10 trials of 10 subjects (*n* ≥ 100) each for each clinical study. Specifically, a two-step validation process for the FLU PBPK/PD model was followed. The initial model was internally verified by comparing the predicted and observed plasma concentration profiles for the IV and the oral solution (67.9 mg) administrations. The model was then validated by comparing mean simulated and observed plasma concentration profiles, and exposure and response parameters of external datasets including PK data from subjects with different CYP2C9 genotypes in a 40–300 mg dose range. Virtual populations were selected to closely match the enrolled individuals in the respective *in vivo* clinical trials with regard to sample size, ethnicity, gender ratio, and age and weight range. Reported volumes of concomitant liquid intake, dosage form type, and sampling schedule were also included in the study design. A schematic of the modeling workflow is presented in Figure 1.

The predictive performance of the model was assessed by visual predictive checks (5th and 95th percentiles), as well as by comparing predicted and observed plasma concentration values and PK parameters: maximum plasma concentration (C_max_), area under the curve extrapolated to infinity (AUC_inf_) and apparent clearance (CL/F). For this purpose, the ratio (R_pred/obs_) of model-predicted versus observed parameter values was determined (R_pred/obs_ = model-predicted/clinically observed). The predictive accuracy was evaluated on the basis of the “two-fold” rule (−0.301 < logR_pred/obs_ < 0.301), as well as the more stringent deviation of 25% (−0.097 < logR_pred/obs_ < 0.097).

As quantitative measures of model performance, mean relative deviations (MRDs) of the predicted plasma concentrations and geometric mean fold errors (GMFEs) of C_max_, AUC_inf_, and CL/F were also calculated, as follows:

(4) $$MRD=10^{\frac{1}{N}\sqrt{\overset{N}{\underset{i}{\sum }}{\left(log_{10}\left(C_{i}\right)-log_{10}\left(\hat{C_{i}}\right)\right)}^{2}}}$$

(5) $$GMFE=10^{\frac{1}{n}\overset{n}{\underset{j}{\sum }}\left|log_{10}\left(\frac{\hat{a_{j}}}{a_{j}}\right)\right|}$$

where $C_{i}$ and $\hat{C_{i}}$ are the $i^{th}$ observed and predicted concentrations, respectively;$\;a_{j}$ and $\hat{a_{j}}$ correspond the observed and the respective predicted *C_max_, AUC_inf_*, or *CL/F* values of the $j^{th}$ clinical study; and *N* and *n* are the number of observations and clinical studies, respectively. Overall MRD and GMFE values of ≤2 were considered as reasonable predictions [75,76,77].

#### 2.9.4. PBPK DDI Modeling

In addition to the evaluation methods described in Section 2.9.3, we simulated CYP2C9 drug–drug–gene interactions to evaluate the DDI performance of the developed PBPK/PD model. A total of 12 plasma concentration–time profiles after co-administration of flurbiprofen with the strong CYP2C9 inhibitor fluconazole and 1 with the CYP2C9 inducer rifampicin were used to predict the drug–drug–gene interactions of flurbiprofen.

##### PBPK Models of Perpetrator Drugs

The compound files for fluconazole (inhibitor) and rifampicin MD (inducer) are available in the Simcyp (v18.2) drug library, and the verified built-in values for the inhibition and induction parameters were used for these perpetrator drugs. For the clinical trial simulation, the administration protocol and the virtual subjects closely matched the ones from the actual studies.

##### PBPK DDI Modeling Evaluation

The model performance in predicting the DDIs was evaluated by comparison of the predicted to observed victim drug plasma concentration–time trajectories, when administered alone and during co-administration. The ratios of AUC from time zero to the time of the last measured concentration (AUC_last_) and of C_max_, with and without administration of the perpetrator drug, were calculated as follows:

(6) $$DDI\;ratio=\frac{AUC_{last}\;or\;C_{max}\;victim\;drug\;during\;perpetrator\;coadministration\;}{AUC_{last}\;or\;C_{max}\;victim\;drug\;\left(control\right)}$$

To assess the DDI modeling, the GMFEs of the predicted DDI were calculated for AUC_last_ and C_max_ ratios according to Equation (5).

#### 2.9.5. Virtual Populations

North European Caucasian (NEurCaucasian) and Chinese virtual populations of healthy volunteers were used for the population simulations of this study. The main differences in the inputs for the two populations related to CYP2C9 metabolism and genotype profile are summarized in Table S1. The intrinsic catalytic activity of CYP2C9 per unit amount of enzyme variant and tissue composition were assumed the same in both populations. The mean default intestinal and liver CYP2C9 abundances as well as the specific genotype frequencies of the Simcyp population libraries were used. As the Korean population is not available in the current Simcyp version (v19.1), we simulated studies including Korean subjects by using the Chinese virtual population, which is considered to be the population with the highest demographic and genetic proximity to the Korean population [78,79,80].

### 2.10. Data Analysis and Model Diagnostics

The solubility and dissolution data are presented as the arithmetic mean (standard deviation). Model-based analysis of the *in vitro* data in the SIVA Toolkit was performed with either Nelder–Mead or a hybrid algorithm with a 5th order Runge–Kutta solver. The appropriate weighting scheme was chosen on the basis of the observed data ranges and their homogeneity, and the goodness of fit was assessed by the coefficient of determination (*R^2^*) as well as visual predictive checks (e.g., residuals plots). All PK profiles obtained from the literature were digitalized with the WebPlotDigitizer (version 4.1; PLOTCON; Oakland, USA). The parameter estimation within the PE module of the Simcyp Simulator was performed with the maximum likelihood estimation method.

Data post-processing and visualization were performed with MATLAB 2019b (Mathworks Inc.; Natick, MA, USA) and R version 3.5.3 (R Core Team (2019). R: A language and environment for statistical computing. R Foundation for Statistical Computing, Vienna, Austria. URL https://www.R-project.org/).

## 3. Results

### 3.1. In Vitro Solubility

Table 4 summarizes the equilibrium solubility values in multiple aqueous buffers and biorelevant media with different pH values. The final pH_bulk_ differed significantly from the initial pH values in phosphate buffers of different pH values due to the self-buffering effect. In fact, the reduction is even more pronounced in the fasted state biorelevant media due to their lower buffer capacity (5.6 mmol/L/ΔpH in FaSSIF V3 versus 18.5 mmol/L/ΔpH in European Pharmacopoeia phosphate buffers) [24]. Such a behavior was not observed for the FaSSGF Level I and III, the acetate buffer, and the FeSSIF Level I, where the respective pH change was limited to 0.1 pH unit.

Micelle-mediated solubilization seemed not to have a substantial impact on the overall solubility of FLU, which is instead highly dependent on pH.

### 3.2. Model-Based Analysis of In Vitro Solubility Data

Table 5 summarizes the parameter estimates (95% CI) obtained by model-based analysis of the *in vitro* solubility data in compendial and biorelevant media, as described in Section 2.6. The pK_a_ was determined to be 4.05, a value which agrees with values reported in the literature [54,57,60,81]. By estimating the micelle-water partition coefficients for both neutral and ionized species using the biorelevant solubilities, we were able to quantify the effect of physiologically relevant surfactants on the overall solubility of FLU. These values were used as inputs to the Simcyp Simulator (Table 3) to simulate luminal conditions and the *in vivo* dissolution behavior, accounting at the same time for inter-subject variability regarding bile salt-mediated solubilization in the virtual population. Therefore, implementation of logK_m:w_ values for the nonionised (“neutral”) and ionised forms of FLU in the PBPK model enabled mechanistic prediction of the *in vivo* luminal dissolution population variability, which would not be possible if only mean solubility values had been used.

### 3.3. In Vitro Dissolution Tests

**Figure 2:**
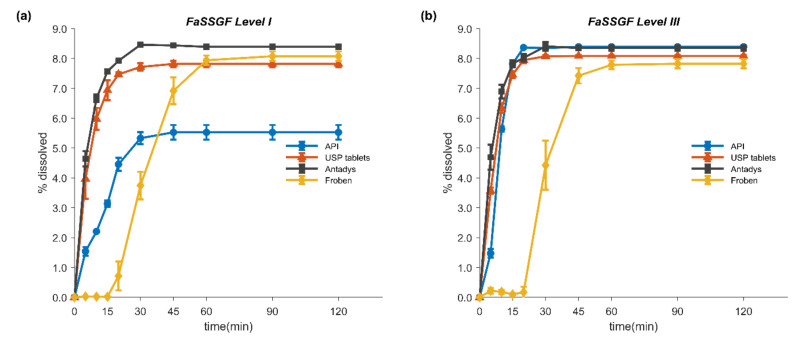
*In vitro* dissolution (mean ± SD) of flurbiprofen active pharmaceutical ingredient (API) 100 mg (circles), FLU USP tablets 100 mg (triangles), Antadys 100 mg (squares), and Froben 100 mg (diamonds) in fasted state-simulated gastric fluid (FaSSGF) Levels I (**a**) and III (**b**), respectively. USP paddle apparatus at 75 rpm and 250 mL of dissolution medium at 37°C were used in all experiments. All experiments were performed at least in triplicate (*n* ≥ 3). Most standard deviation bars lie within the symbols.

Figure 2 shows the mean percentage dissolved (± SD) of FLU in the tested formulations and as pure drug over time in fasted state simulated gastric fluids (FaSSGF) of different simulation levels (I and III). As expected, the *in vitro* release of this poorly soluble weak acid under gastric conditions was incomplete, reaching a plateau at around 8.3% of the dose in both FaSSGF Levels I and III. The USP as well as the Antadys tablets exhibited similar *in vitro* dissolution behavior in both media. However, the unformulated drug reached a maximum of only 5.5% in FaSSGF Level I. Since there was no difference in the solubility of FLU between the two media, this observation was attributed to the absence of surfactants and proteins (i.e., pepsin) in FaSSGF Level I, leading to poor wetting of the drug powder.

**Figure 3:**
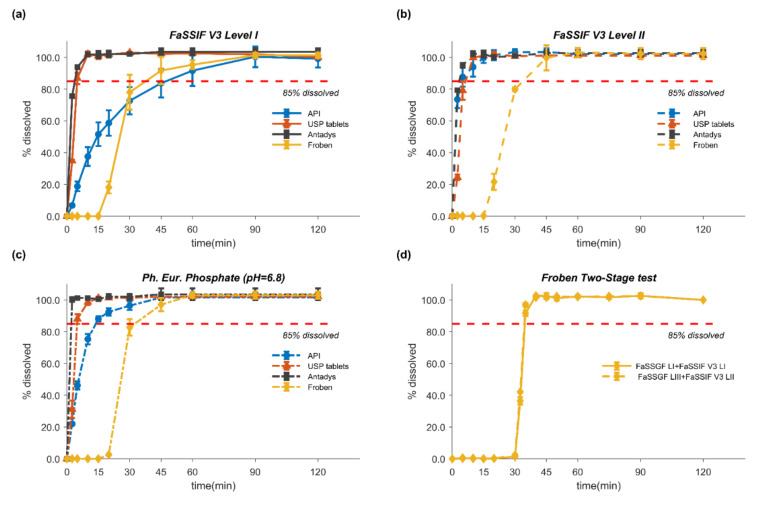
*In vitro* dissolution (mean ± SD) of flurbiprofen API 100 mg (circles), FLU USP tablets 100 mg (triangles), Antadys 100 mg (squares), and Froben 100 mg (diamonds) in (**a**) FaSSIF V3 Level I (solid lines), (**b**) FaSSIF V3 Level II (dashed lines), and (**c**) Ph. Eur. phosphate buffer (pH = 6.8) (dashed dotted lines). (**d**) Two-stage test of Froben 100 mg (diamonds) in FaSSGF Levels I (solid line) and III (dashed line) at the gastric and FaSSIF V3 Level I (solid line) and FaSSIF V3 Level II (dashed lines) at the intestinal compartments, respectively. USP paddle apparatus at 75 rpm at 37 ± 0.4 °C was used in all experiments. The volume of dissolution medium in the gastric compartment was 250 mL, to which 250 mL of appropriately concentrated intestinal medium was added after 30 min. Horizontal dashed red lines represent the 85% dissolved. All experiments were performed at least in triplicate (*n* ≥ 3). Most standard deviation bars lie within the symbols.

Mean percentage dissolved (± SD) over time in compendial and fasted state-simulated intestinal fluids (FaSSIF) for the unformulated API and the tested formulations are presented in Figure 3a–c. For the pure drug, the dissolution in FaSSIF V3 Level II and in Ph. Eur. phosphate buffer (pH 6.8) was very rapid (>85% within 2.5 and 15 min, respectively). On the other hand, dissolution in FaSSIF V3 Level I (i.e., without bile components) was much slower, with 85% dissolved reached only after 60 min. Such behavior can be assigned to differences in buffer capacity (FaSSIF V3 Level I and II vs. phosphate buffer), solubilization capacity (FaSSIF V3 Level II vs. Level I), and wettability of the tested media. The difference of 0.1 pH units between the initial pH of Ph. Eur. phosphate buffer (pH 6.8) and FaSSIF V3 is assumed to have had a negligible effect.

Especially since dissolution was performed under non-sink conditions in this series of experiments, the dissolution rate of the pure drug in FaSSIF V3 Level I was significantly slower, due to its low buffer capacity (5.6 mEq/L/ΔpH), than in the compendial 50 mM phosphate buffer (25 mEq/L/ΔpH) [82]. At the higher total phosphate buffer concentration of the compendial medium (50 mM), the bulk (pH_bulk_) rather than the surface pH (pH_0_) drove solubility and dissolution. By contrast, in the low buffer capacity FaSSIF V3 Level I medium, the surface pH seemed to control the dissolution rate. Indeed, the influence of the dissolving acid on the medium was so great that even the bulk pH was significantly altered (final pH was 6.31 vs. 6.82 in Ph. Eur. phosphate buffer). The self-buffering effect on the overall dissolution behavior was much less prominent when bile salts were added to the medium, as shown in Figure 3b. Furthermore, it was evident that the addition of the bile salt components in FaSSIF V3 Level II markedly enhanced the dissolution rate of the unformulated FLU. Although the main effect was likely through solubilization, improvements in wetting seemed to have also contributed to the higher dissolution rate in the Level II medium, given that a similar behavior was observed in the gastric media.

For the USP tablets and Antadys, these trends were not observed, and dissolution was very fast (85% dissolved within 10 min) in all tested “intestinal” media. Interestingly, Froben, the sugarcoated formulation, consistently showed long disintegration times, with no dissolution for up to 20 min. irrespective of the pH, buffer capacity, or the inclusion of bile salt components in the medium. These findings suggest that Froben would be classified as slowly dissolving if the formulation was solely exposed to the intestinal media without considering the disintegration of the sugar coating in the stomach. In order to account for disintegration in the stomach prior to exposure to the intestinal media, we performed two-stage dissolution tests (Section 2.4). The results from the two-stage tests (Figure 3d) revealed that as long as disintegration takes place in the gastric compartment, the dissolution from Froben tablets in the intestinal medium is very fast, reaching 85% dissolved within 5 min.

### 3.4. Modeling of In Vitro Dissolution

Table 6 and Table 7 summarize the estimated DLM scalar values (95% CIs) obtained by model-based analysis of the gastric and intestinal *in vitro* dissolution profiles using the SIVA Toolkit. The goodness of fit was visually inspected with residual plots and assessed with the coefficient of determination (*R^2^*). As shown in Table 6, the slowest dissolution rate of the API observed in FaSSIF V3 Levels I and the fastest of Antadys in FaSSIF V3 Level II resulted in the lowest (0.00185) and highest (0.0125) estimated DLM scalar values (S_DLM_), respectively. Differences in the S_DLM_ estimates of the gastric dissolution were not expected to have a major impact on the *in vivo* performance of FLU since the release in the stomach is very poor.

Given the high solubility of FLU in intestinal media, we expected disintegration rather than API solubility to be the rate-limiting step for the dissolution rate of Froben. In this context, all intestinal single-stage dissolution profiles of Froben can be modelled by a universal first-order disintegration rate constant and a lag time in dissolution. Alternatively, modeling of the profiles obtained from the two-stage tests as serial dilutions of different media should be a more physiological approximation of the gastrointestinal (GI) luminal conditions. The estimates from both approaches are presented in Table 7.

In a dissolution-based *in vitro–in vivo* extrapolation (IVIVE) approach, the gastric and intestinal DLM scalar (S_DLM_) estimates were transferred to the Simcyp simulator to generate medium-customized and formulation-specific *in vivo* dissolution scenarios and to simulate FLU *in vivo* performance.

All fitted dissolution profiles were in excellent agreement with the experimental ones with *R^2^* > 0.94.

### 3.5. PBPK/PD Model Development and Evaluation

**Figure 4:**
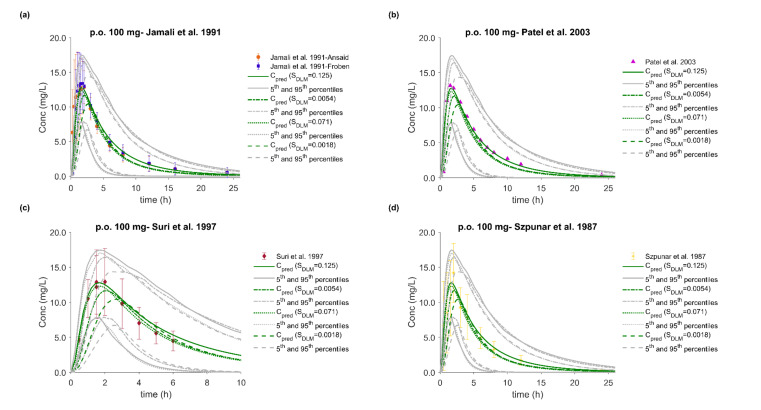
Mean flurbiprofen plasma concentration–time profiles after oral administration of 100 mg tablet in healthy Caucasians. Population simulations (*n* = 100) under four *in vivo* dissolution scenarios are shown as green and grey lines for the mean and the 5th and 95th percentiles, respectively. Each dissolution scenario is represented by the corresponding S_DLM_ value and is shown with different line style: S_DLM_ = 0.125 (solid line), S_DLM_ = 0.071 (dotted line), S_DLM_ = 0.0054 (dashed-dotted line), and S_DLM_ = 0.0018 (dashed line). Observed data with SD, if available, are depicted as (**a**) circles (Jamali et al., Ansaid) and squares (Jamali et al., Froben); (**b**) triangles (Patel et al.); (**c**) diamonds (Suri et al.); (**d**) asterisks (Szpunar et al.). References link to a specific observed dataset described in Table 1.

**Figure 5:**
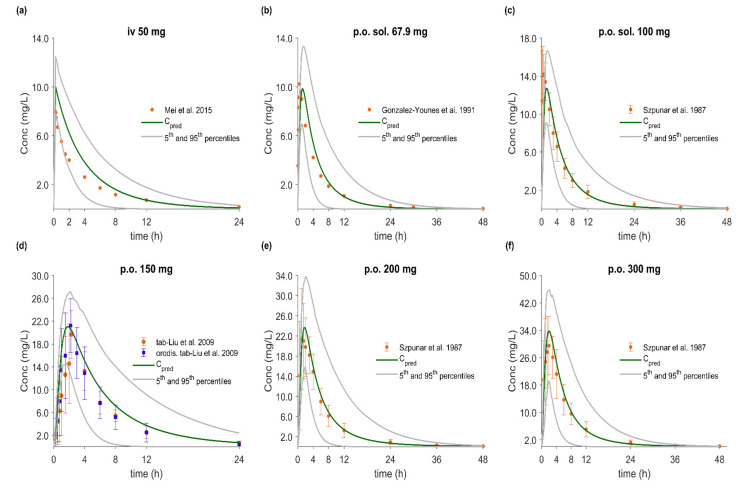
Mean flurbiprofen plasma concentration–time profiles after intravenous and oral administration in healthy Chinese (**a**,**d**) and Caucasian (**b**,**c**,**e**,**f**) individuals. Population simulations (*n* = 100) are shown as green and grey solid lines for the mean and the 5th and 95th percentiles, respectively. Observed data with SD, if available, are depicted as circles and squares. References link to a specific observed dataset described in Table 1. Administration protocol: (**a**) 50 mg intravenously; (**b**) 67.9 mg oral solution; (**c**) 100 mg oral solution; (**d**) 150 mg oral tablet; (**e**) 200 mg oral tablet; (**f**) 300 mg oral tablet.

**Figure 6:**
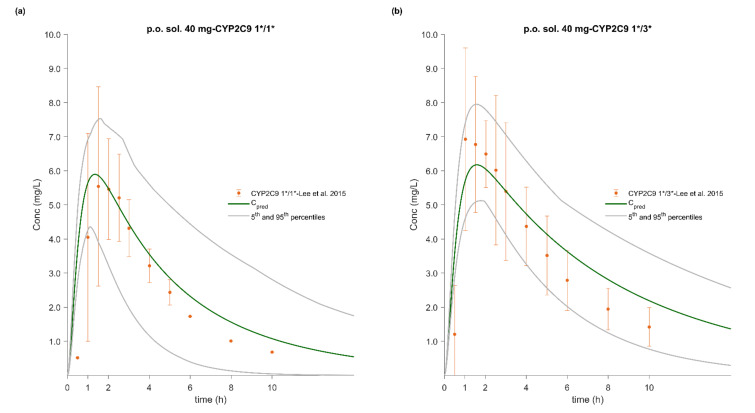
(**a,b**) Mean flurbiprofen plasma concentration–time profiles after administration of 40 mg oral solution in CYP2C9 1*/1* and 1*/3* healthy Korean volunteers, respectively. Population simulations (*n* = 100) are shown as green and grey lines for the mean and the 5th and 95th percentiles, respectively. Observed data, with SD, are depicted as circles. References link to a specific observed dataset described in Table 1.

**Figure 7:**
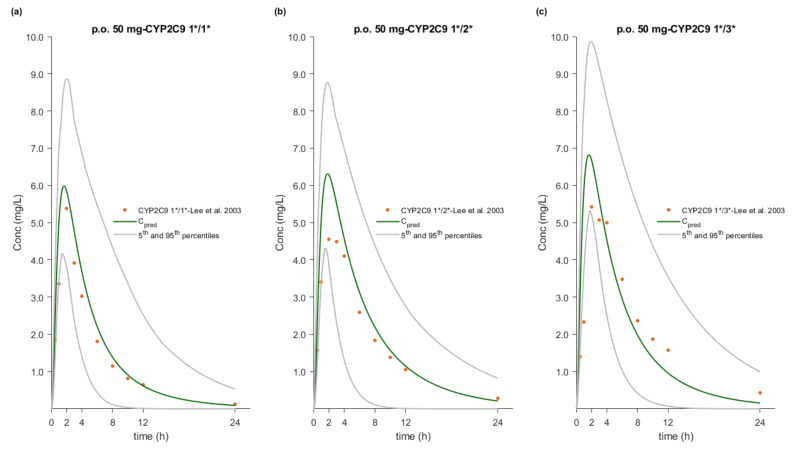
(**a**–**c**) Mean flurbiprofen plasma concentration–time profiles after administration of 50 mg oral tablet in CYP2C9 1*/1*, 1*/2*, and 1*/3* healthy Caucasian volunteers, respectively. Population simulations (*n* = 100) are shown as green and grey lines for the mean and the 5th and 95th percentiles, respectively. Observed mean data are depicted as circles. References link to a specific observed dataset described in Table 1.

The whole-body PBPK model of FLU accurately described and predicted plasma concentration–time profiles following intravenous and oral administration over a wide dose range (Figure 4, Figure 5, Figure 6 and Figure 7). For the development and validation of the PBPK model, we used 17 plasma concentration–time profiles, including 5 for subjects with specific CYP2C9 genotypes. *In vitro* dissolution data available for the 100 mg immediate release solid oral products were modelled and incorporated into the PBPK model to simulate various *in vivo* dissolution scenarios. At any other dose level, including the CYP2C9 polymorphism studies, we used the fastest dissolution rate (S_DLM_ = 0.125) as input. When the administered form was an oral solution, we considered the entire dose to be pre-dissolved.

The predictive performance of the PBPK model is demonstrated via visual comparisons of predicted versus observed plasma concentration–time profiles as well as quantitative measures such as MRDs and GMFEs. The predictions of plasma concentration–time trajectories for all routes of administration, doses, and drug products are in close agreement with the observed data. Applying a twofold deviation as the upper limit for an adequate prediction, the PBPK models achieved 100% ability to predict AUC_inf_, C_max_, and CL/F adequately. When a more stringent acceptance criterion (i.e., 25% deviation) was applied, the predictions of AUC_inf_, C_max_, and CL/F were adequate in 90%, 81%, and 74% of the cases, respectively. Moreover, the MRD values were within twofold in 94% of the studies, with only about 20% less than 1.25-fold. The overall MRD values for the FLU PBPK model and GMFE values for AUC_inf_, C_max_, and CL/F were 1.59 (1.04–2.43), 1.14 (1.00–1.39), 1.15 (1.01–1.41) and 1.18 (1.06–1.39), respectively. Detailed results along with calculated MRD and GMFE values for all studies are presented in Table 8 and Table 9.

**Figure 8:**
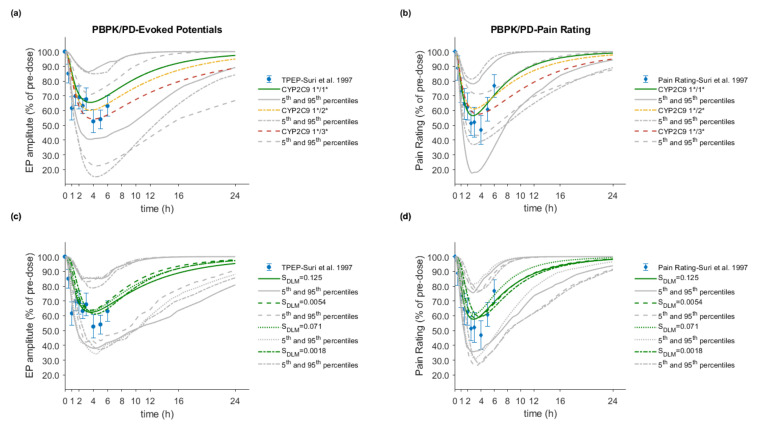
Mean flurbiprofen response time profiles after administration of 100 mg oral tablet in healthy Caucasians. (**a**,**b**) Genetic polymorphism: population simulations (*n* = 100) in CYP2C9 1*/1*. 1*/2* and 1*/3* are shown for the mean as green (solid), yellow (dash dotted), and orange (dashed) lines, respectively. Grey lines with the corresponding style represent the 5th and 95th percentiles. (**c**,**d**) Dissolution rate: Population simulations (*n* = 100) under four *in vivo* dissolution scenarios are shown as green and grey lines for the mean and the 5th and 95th percentiles, respectively. Each dissolution scenario is represented by the corresponding S_DLM_ value and is shown with different line style: S_DLM_ = 0.125 (solid line), S_DLM_ = 0.071 (dotted line), S_DLM_ = 0.0054 (dashed line), and S_DLM_ = 0.0018 (dashed-dotted line). Observed data with SD, if available, are depicted as circles. References link to a specific observed dataset described in Table 1.

The final PBPK model was further coupled with a PD FLU analgesic efficacy model. The integrated PBPK/PD model was able to capture the pain-relieving response of S-FLU after oral administration of 100 mg racemic FLU. The predictive performance was assessed by comparing the predicted with the observed response time profiles for two PD endpoints, the TPEP amplitude and pain rating (see Figure 8). Regardless of the *in vivo* dissolution rate or the genotype of the virtual individuals, the predictive accuracy for the prediction of the PD metrics, maximum response (R_max_), time to maximum response (TR_max_), and area under the effect-time curve (AUCE), was in all in cases within 1.25-fold (see Table S2).

### 3.6. Effect of Dissolution Rate

Several *in vitro* dissolution profiles from various marketed FLU immediate release oral products at the highest dose strength of 100 mg and under different *in vitro* conditions were generated. In a dissolution-based IVIVE approach, and after modeling of the *in vitro* data using the diffusion layer model, the obtained S_DLM_ values (for stomach and small intestine) were integrated into the PBPK/PD model to investigate the impact of different *in vivo* dissolution rates on the PK/PD of FLU. Population simulations (*n* = 100) were performed with the NEurCaucasian virtual population and the enzymatic status of each virtual subject was tracked. The overall mean predicted plasma concentration–time profiles of each dissolution scenario were compared with observed PK profiles from five external datasets and among the datasets (Figure 5). Between the results of the fastest (S_DLM_ = 0.125) and slowest (S_DLM_ = 0.0018) dissolution rates (corresponding to 85% dissolved under intestinal conditions in 2.5 and 60 min, respectively) a decrease of only about 20% in both C_max_ and AUC_inf_ was observed (Table 9). On the other hand, t_max_ was prolonged by 30 min (data not shown). Despite these differences, in all cases, the predictive accuracy was acceptable with MRD between 1.04 and 2.43 and GMFE values ranging from 0.78 to 1.01 for C_max_ and 0.76 to 1.20 for AUC_inf_.

The PD metrics, R_max_, and AUCE of any of the two endpoints were not noticeably affected by the *in vivo* dissolution rates. Any previous discrepancies in the PK parameters (C_max_ and AUC_inf_) did not translate to differences in R_max_ and AUCE, rather, they were mitigated to less than 5.5% and 7%, respectively. However, the TR_max_ was prolonged by up to 1h when the slowest dissolution rate was applied, indicating a potential clinical relevance of slow dissolution on the onset and the time to maximum analgesic action. Simulations of the response time profiles and comparison with the actual clinical data for each dissolution rate and for both endpoints are depicted in Figure 8c,d. Detailed results for the PD together with the calculated R_pred/obs_ are shown in Table S2.

### 3.7. Effect of CYP2C9 Genetic Polymorphism

PBPK simulations accurately captured the observed effect of three different CYP2C9 genotypes on FLU PK in Caucasian and Chinese populations. Population simulations (*n* = 300) were performed using the NEurCaucasian and Chinese virtual populations to reproduce the clinical studies published by Lee et al. (2003) and Lee et al. (2015), respectively [37,42]. The sample size in these population simulations was increased to 300 (30 trials of 10 subjects each) to ensure adequate representation of each genotype. The enzymatic status of each virtual subject was tracked, and the individual plasma concentration–time profiles were stratified on the basis of the CYP2C9 genotype. The range of GMFE values for C_max_, AUC_inf_, and CL/F was 0.90–1.39, 0.91–1.12, and 0.76–1.03, respectively. An overall reduction of 42% and 38% in the clearance of CYP2C9 1*/3* individuals of both populations, which in turn led to a 1.52- and a 1.62-fold increase in AUC, respectively, was predicted. These findings are in close agreement with the observed data from Lee et al. (2003) and Lee et al. (2015), who reported a decrease in CYP2C9 1*/3* clearance of about 37% and 44%, resulting in a 1.62- and 1.74-fold increase in AUC, respectively. The genotypes and study specific MRDs and GMFEs are summarized in Table 8 and Table 9.

The model was used to simulate the response time curves of subjects with specific CYP2C9 genotypes (1*/1*, 1*/2*, and 1*/3*) in order to explore potential PD differences. Population simulations showed no effect on R_max_ and TR_max_, whereas a 1.35-fold increase in the AUCE for the CYP2C9 1*/3* subjects was predicted using the TPEP amplitude as the endpoint (Figure 8a,b). However, when the subjective pain rating score scale was used, no consistent increase in the AUCE was observed. Interestingly, in comparison to the wild type (CYP2C9 1*/1*), the time post-administration to return to 80% of the initial value (*T_80% initial_*) in 1*/3* subjects was delayed by about 7 and 4.5 h for both TPEP and pain rating, respectively. A similar but less pronounced effect was also predicted for the 1*/2* subjects. Details of the simulation results together with the R_max_, TR_max_, AUCE, and *T_80% initial_* exact values are summarized in Table S2.

### 3.8. Drug–Drug–Gene Interactions

A total of 13 sets of plasma concentration–time profiles were available in the literature for evaluation of model-predicted interactions. Accurate prediction of the impact of a perpetrator on the pharmacokinetics of a victim drug ratifies the capacity of the victim drug PBPK model to correctly predict the amount of drug eliminated via the affected pathway and indicates that the perpetrator model properly describes the concentration of the inhibitor/induced at the site(s) of interaction. Furthermore, accurately capturing not only drug–drug but also drug–gene interactions reinforces the model confidence in describing the effect of genotype on the pharmacokinetics of the substrate drug.

**Figure 9:**
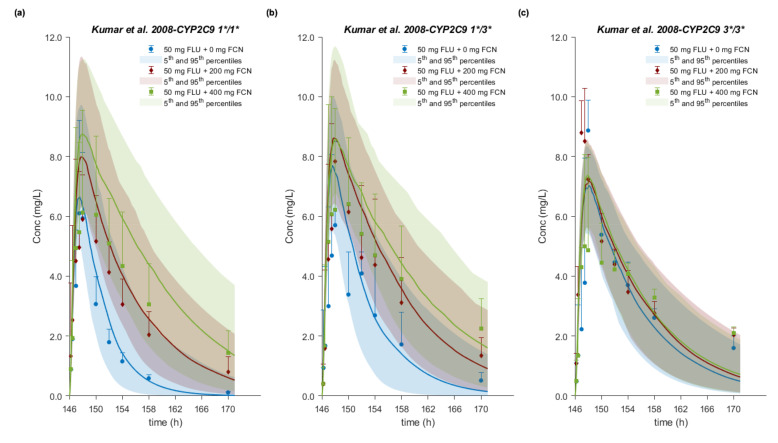
**(a**–**c)** Mean plasma concentration–time profiles after administration of 50 mg flurbiprofen as oral tablet alone and with 200 mg or 400 mg fluconazole (FCN) in CYP2C9 1*/1*, 1*/3*, and 3*/3* healthy Caucasian volunteers, respectively. Population simulations (*n* = 100) are shown for the mean as blue (FLU + 0 mg FCN), red (FLU + 200 mg FCN), and light green (FLU + 400 mg FCN) solid lines, and observed data with SD, if available, are depicted as circles, diamonds, and squares, respectively. Shaded areas represent the 5th and 95th percentiles. References link to a specific observed dataset described in Table 2.

In subjects with three CYP2C9 genotypes, the wild type and both hetero- and homozygotes for the CYP2C9*3 allele, the PBPK model successfully predicted the gene dose-dependent interactions with the prototype moderate inhibitor (fluconazole). The AUC ratio was slightly underpredicted in 1*/1* and 1*/3* subjects at the 400 mg fluconazole dose level (R_pred/obs_ = 0.74–0.78). Nevertheless, the concentration time course of the victim drug with and without coadministration at both inhibitor dose levels and for all genotypes was accurately captured (Figure 9).

**Figure 10:**
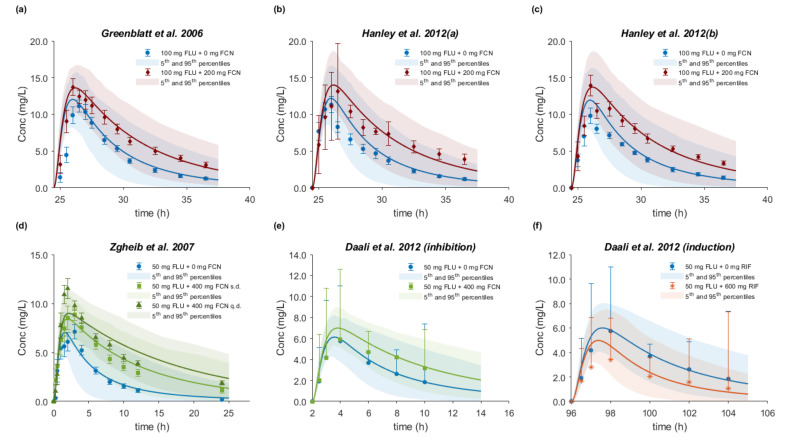
Mean plasma concentration–time profiles after administration of flurbiprofen alone and with the perpetrator drug in healthy volunteers. (**a**–**e**) Population simulations (*n* = 100) without or with the CYP2C9 inhibitor fluconazole (FCN) are shown for the mean as blue (FLU + 0 mg FCN), red (FLU + 200 mg FCN), light green (FLU + 400 mg FCN s.d.), and dark green (FLU + 400 mg FCN q.d.) solid lines, and observed data with SD, if available, are depicted as circles, diamonds, squares, and triangles, respectively. (**f**) Population simulations (*n* = 100) without or with the CYP2C9 inducer rifampicin (RIF) are shown for the mean as blue (FLU + 0 mg RIF) and orange (FLU + 600 mg RIF) solid lines, and observed data with SD, if available, are depicted as circles and asterisks, respectively. Shaded areas represent the 5th and 95th percentiles. References link to a specific observed dataset described in Table 2.

PBPK model simulations successfully predicted the FLU–fluconazole interaction under different dose levels and regimens in six clinical studies, in which no prior genotyping had been performed (Figure 10a–e). All DDI AUC, C_max_, and CL/F ratios were within 1.25-fold. The rifampicin inductive effect on the exposure of FLU was also accurately predicted from one study, with DDI ratios within 1.25-fold (Figure 10f).

The DDI predictive accuracy was further evaluated by calculation of the GMFE values for the DDI AUC, C_max_, and CL/F ratios, which ranged from 1.15 to 1.17. The corresponding R_pred/obs_ values for DDI AUC, C_max_, and CL/F ratios of all modeled DDI studies together with the GMFEs are listed in Table 10.

## 4. Discussion

In the present study, we developed a comprehensive PBPK/PD model of FLU, which allows for consistent and accurate representation of the dose–exposure relationship after intravenous and oral administration of different dosage forms in Caucasian and Chinese healthy populations over a wide dose range (40–300 mg). The model mechanistically describes the absorption and precisely predicts the impact of formulation and dissolution rate on the PK of FLU. By integrating *in vitro* metabolism with demographic and *in vivo* data, the model is able to quantify the contribution of the CYP2C9 polymorphic alleles on the elimination pathways, providing gainful insight into the magnitude of genetic polymorphism on the pharmacokinetic behavior of FLU. By linking the verified PBPK model with an inhibitory *E_max_* model describing the analgesic efficacy of the drug, we used the final PBPK/PD model to explore the effect of system or extrinsic factors on the onset and duration of pain-relieving action as well as to suggest dose adjustments for specific genetic sub-populations. Furthermore, the PBPK model successfully predicted gene dose-dependent DDIs, allowing for dose optimization recommendations, increasing confidence in the predictive accuracy and robustness of the model.

High inter-individual variability in PK studies of FLU has been associated with complex and variable oral absorption, including double peak phenomena [2]. This variability, often reaching up to 100%, is consistent among studies with respect to both C_max_ and AUC. As a typical BCS class II weak acid, flurbiprofen absorption from the small intestine is expected to be dissolution-limited and therefore the formulation and its dissolution rate will be critical to the *in vivo* performance. At the same time, FLU is mainly eliminated (>71% of the dose) through metabolic oxidation to its primary metabolite, 4-hydroxy FLU, exclusively by the P450 CYP29. As a result, FLU exhibits polymorph-dependent PK, which is affected by concomitant administration of CYP2C9 inhibitors such as fluconazole. Although FLU has been extensively studied and has been recommended as probe drug for CYP2C9 substrates, relatively few studies have been published regarding its PK/pharmacogenomic and clinical interactions [43,44,45,46,47].

The present PBPK/PD model leveraged data from multiple *in vitro* sources and *in vivo* human studies. Prior to model development, we performed a careful biopharmaceutic analysis, including formulation selection, biorelevant *in vitro* solubility, and dissolution experiments. Data analysis of the *in vitro* results enabled translation and extrapolation of the biopharmaceutic parameters to the *in vivo* luminal conditions, providing mechanistic insight into the oral absorption of the drug. The initial PBPK model was informed with allele-specific *in vitro* metabolism data to account for differences in the elimination, due to CYP2C9 genetic polymorphism, and successfully predicted (36 out of 38) observed concentration–time profiles and CYP2C9 genetic effects within a predefined twofold deviation boundary (Table 8). In both cases where MRD fell outside the twofold limit, the slowest dissolution rate, corresponding to 85% release only after 60 min, was used as the input profile and resulted in sub-optimal absorption and underprediction of C_max_ and AUC.

For further evaluation and enhanced prediction accuracy, we implemented a more stringent success measure consisting of a 25% deviation boundary. This predefined criterion is not meant to be equated to the bioequivalence acceptance limits (i.e., 80–125%), but rather is selected to be sufficiently conservative to prevent poor decision-making due to misclassified predictions. All individual model predictions for the pharmacokinetic parameters C_max_, AUC_inf_, and CL/F were within twofold, and 90%, 81%, and 74% of them, respectively, satisfied the 25% deviation criterion. The slight underprediction (*R_pred/obs_ = 0.76–0.78)* of C_max_ and AUC in the Jamali et al. study after oral administration of 100 mg was associated with the input of the slowest intestinal dissolution rate (S_DLM_ = 0.0018) [38]. By contrast, the C_max_ was overpredicted (*R_pred/obs_ = 1.36–1.39)* in CYP2C9 1*/3* individuals at 50 and 150 mg. Deviations from the 1.25-fold boundary in AUC (*R_pred/obs_ = 0.71–0.78)* and clearance (*R_pred/obs_ = 1.31–1.39)* were consistently predicted under all dissolution scenarios, except the slowest (S_DLM_ = 0.0018), when simulating the study by Patel et al. [4]. Nevertheless, it has to be noted that in most studies, the participants were not subjected to prior genotype screening, and only the mean plasma concentration–time profiles were reported.

Population simulations, after translation of *in vitro* release into *in vivo* dissolution rates, provided insight into the impact of absorption variables on FLU PK/PD. Interestingly, it was shown that differences between the fastest (85% dissolved in 2.5 min) and the slowest (85% dissolved in 60 min) *in vivo* dissolution rates (S_DLM_ = 0.0018 vs. S_DLM_ = 0.125) translated into a decrease in C_max_ of only approximately 20%, while t_max_ was prolonged by 30 min. These simulations indicate that *in vitro* dissolution rate might not be the most critical attribute for the *in vivo* performance. Instead, they suggest that the interplay between absorption and metabolism plays a key role, given also that flurbiprofen’s half-life is rather short (3–7 h). Regardless of the shift in the regional absorption peak from mid-jejunum at the fastest dissolution rate to the ileum at the slowest dissolution rate, we predicted the absorption to be complete (f_a_ > 0.93). These (minor) differences in C_max_ and AUC_inf_ did not result in a similar degree of change in R_max_ and AUCE. In fact, they were mitigated to less than 7%, showing that *in vivo* dissolution rate has no or little effect on the degree and duration of analgesic effect. However, at the slowest dissolution rate, the TR_max_ was prolonged to 1h. These findings suggest that t_max_ might be not only a more sensitive metric in single-dose bioequivalence studies of FLU, but also more relevant for the onset of pain relief.

As a probe substrate of CYP2C9, FLU exhibits gene-dependent pharmacokinetics [47,83]. The PBPK model accurately predicted the impact of the three main CYP2C9 polymorphisms on the exposure of the drug in both Caucasian and Chinese healthy volunteers. Model predictions were within 1.25-fold for both AUC (0.91–1.12) and oral clearance (0.76–1.03), while C_max_ was only slightly overpredicted (up to 1.39-fold). These results further increased confidence in the validity of the allele-specific *in vitro* data and added to the overall model robustness. The observed decrease of about 27% and 40% in the clearance of CYP2C9 1*/2* and 1*/3* individuals, respectively, might need to be considered in terms of adjustments to the recommended dose of flurbiprofen. These findings are in agreement with a large genotype–phenotype correlation clinical study, in which the CYP2C9 genotype of 283 healthy subjects was correlated with the metabolic ratio of FLU, calculated from urine data, as the phenotypic metric [84]. In this study, the recommended dose for CYP2C9 1*/2* and 1*/3* subjects was found to be 84% and 60% of the dose administered to the wild type subjects, respectively. Nevertheless, in terms of pain relief, simulations did not show any differences in R_max_ and TR_max_ among polymorphic subjects. However, the return to 80% of the initial pain value was delayed by up to 7 h in CYP2C9*3 heterozygotes, implying a longer duration of action in those subjects. A similar behavior, but to a lesser extent (delay of up to 4.5 h), was also predicted for the CYP2C9 1*/2* subjects. In any case, potential flurbiprofen dose optimization in CYP2C9 polymorphic subjects should be carefully evaluated under consideration of the exposure–response and exposure–safety relationships.

The present PBPK analysis was extended to simultaneously investigate the effect of genetic polymorphism and perpetrator co-administration on FLU PK by predicting drug–drug and drug–gene interactions. The *R_pred/obs_* of DDI AUC, C_max_, and CL/F ratios from 11 clinical studies with 200 and 400 mg fluconazole (inhibitor) and one with 600 mg rifampicin (inducer) co-administration ranged from 0.74 to 1.43 with GMFE values within 1.25-fold in 8, 9, and 10 out of 12 in total studies, respectively. Only one drug–drug–gene interaction study was available in the literature, in which flurbiprofen alone or together with 200 and 400 mg fluconazole was administered to CYP2C9 1*/1*, 1*/3*, and 3*/3* healthy volunteers [43]. Our model accurately described the plasma concentration–time profiles with and without the inhibitor in all polymorphic groups. On the basis of the *in silico* DDI studies, at a 400 mg dose of fluconazole, we would classify the interaction in 1*/1* (or assuming 1*/1*) subjects as weak/moderate with AUC ratio between 1.53 and 2.87. Interactions at a 200 mg dose of fluconazole and a 600 mg dose of rifampicin would be considered as weak, with AUC ratios of 1.51–1.94 and 0.63, respectively. The interaction for 1*/3* and 3*/3* subjects at 200 mg with AUC ratios 1.58 and 1.09, respectively, and at 400 mg fluconazole, with AUC ratios of 1.84 and 1.16, respectively, was predicted to be weak as well. All these simulated trials are in line with the results from the *in vivo* DDI studies. Interestingly, the flurbiprofen–fluconazole interaction was gene dose-dependent. Virtually no change in the apparent oral clearance occurring in 3*/3* subjects due to the already reduced CYP2C9 activity was observed, and despite the very limited number of subjects (*n* = 2), this was also correctly predicted, indicating excellent model performance. From population simulations, a dose reduction of 34–38% in 1*/3* and 60–70% in 3*/3* subjects would be recommended. However, in the case of fluconazole administration, dose adjustments were required for 1*/1* and 1*/3*, but not for 3*/3* individuals.

In drug development and prior to phase II clinical studies, accumulated knowledge regarding the absorption, distribution, metabolism, and excretion attributes of an investigational compound is used for preliminary evaluation of its drug–drug interaction potential. Traditionally, significant exposure changes expected to result from co-medication or genetic polymorphism trigger implementation of dedicated clinical pharmacology studies. Unlike flurbiprofen, most drugs in industry’s contemporary pipelines undergo multiple clearance pathways, and thus exposure variations are expected with co-medication or genetic polymorphism in metabolizing enzymes and/or transporters. In such cases, the clinical trial strategy may not be time- and/or cost-effective and an alternative PBPK/PD modelling approach may be not only more practical, but in some cases indispensable if a wide array of complex drug–drug–gene interactions need to be assessed. The extent and appropriate design of the simulations highly depends on the intended use of the substrate in specific populations, the anticipated co-medications and genetic polymorphisms, the effect of pharmacokinetic changes in safety and efficacy of the drug (e.g., exposure-response relationships), and the design (cohorts, populations, inclusion/exclusion criteria) of prospective DDI or pharmacogenetic studies. In addition, if a drug is known to be subject to a major genetic polymorphism, the European Medicines Agency (EMA) recommends genotyping screening of subjects in exploratory bioavailability studies and all studies using parallel group design, even in crossover bioequivalence studies in case of safety or other pharmacokinetic concerns [85]. In this context, if a translational absorption-modeling framework is established, virtual bioequivalence might be a promising tool as part of the modeling and simulation strategy in both drug and generic drug development. Of course, concerns regarding the impact of genetic polymorphism on the PK/PD can be directly related to the frequency of polymorphic alleles in the population of interest. For example, the frequency of CYP2C9 wild type in Chinese populations is around 97–98%, whereas in Caucasians, approximately 35% of the overall population will have at least one of the CYPC9*2 and/or CYP2C9*3, with an occurrence of 1*/2* and 1*/3* of up to 20% and 10%, respectively [11,86,87]. Thus, genotyping prior to a clinical study of a CYP2C9 substrate in Caucasians might be required, whereas it may be optional in Chinese populations.

## 5. Conclusions

This study highlights the usefulness of translational PBPK/PD modeling and simulation to mechanistically describe the absorption and predict the effect of formulation and CYP2C9 genetic polymorphism on the PK/PD of flurbiprofen. A detailed biopharmaceutic analysis, including appropriately designed biorelevant *in vitro* experiments of various flurbiprofen formulations, was performed initially, followed by *in vitro* data analysis and extrapolation to *in vivo* using a translational framework. Our comprehensive PBPK/PD analyses provided mechanistic insight into the impact of dissolution rate and genotype on the PK/PD. On the basis of these findings, we proposed clinically relevant exposure metric and potential dose adjustments. Furthermore, our PBPK model successfully predicted gene dose-dependent drug–drug interactions, highlighting the robustness of its performance. The present PBPK/PD model could be utilized in future biopharmaceutic applications, dose optimization justifications in healthy population with genetic variations, and PK extrapolations to patient or special populations such as rheumatoid arthritis patients and pediatrics.

Genetic variations and formulation *in vivo* performance appear to be major determinants of individual variability in drug efficacy and safety, representing a challenge in drug development. The translational PBPK/PD approach exemplified in this study attempts to bridge the gap between *in vitro–in silico–in vivo* and allows for accurate and robust clinical predictions tailored to target populations and genotypes, thus paving the way towards personalized medicine.

## Figures and Tables

**Table 1:** Mean (SD) demographic clinical study data used for the development and validation of the physiologically based pharmacokinetic/pharmacodynamic (PBPK/PD) model.

Drug Administration and Formulation	CYP2C9 Genotype	No. of Subjects	Female Ratio	Ethnicity	Age (years)	BW/BW Range (kg)	BH/BH Range (cm)	Reference
**Intravenous**								
50 mg as 10 mg/mL solution (injection within 2 min)	n.a.	24	0	Chinese	-	-	-	Mei et al. [34]
**Oral**								
25 mL of oral solution containing 67.9 mg FLU with 175 mL water;	n.a.	12	0	Caucasian	25–31	-	-	Gonzalez-Younes et al. [35]
40 mL oral solution containing 100 mg FLU with 180 mL water;	n.a.	15	0	Caucasian	29 (18–40)	76.4 (62.3–109.1)	177 (168–188)	Szpunar et al. [36]
Froben solution 40 mg;	1*/1*	12	0	Korean	23.1 (2.4)	65.1 (7.1)	174.8 (5.0)	Lee et al. [41]
Froben solution 40 mg;	1*/3*	8	0	Korean	22 (2.7)	64.6 (7.1)	172.8 (6.4)	Lee et al. [41]
USP tablets (Mylan Pharmaceuticals) 50 mg;	1*/1*	5	0.533	Caucasian	24 (5)	79 (18)	-	Lee et al. [42]
USP tablets (Mylan Pharmaceuticals) 50 mg;	1*/2*	5	0.533	Caucasian	24 (5)	79 (18)	-	Lee et al. [42]
USP tablets (Mylan Pharmaceuticals) 50 mg;	1*/3*	5	0.533	Caucasian	24 (5)	79 (18)	-	Lee et al. [42]
Froben 100 mg with 100 mL water;	n.a.	23	0	Caucasian	27.2 (18–35)	71.8 (52.5)	-	Jamali et al. [38]
Ansaid 100 mg with 100 mL water;	n.a.	23	0	Caucasian	27.2 (18–35)	71.8 (52.5)	-	Jamali et al. [38]
Froben 100 mg with 150 mL water;	n.a.	4	0.5	Caucasian	26.8 (2.2)	67.8 (4.1)	-	Patel et al. [4]
100 mg tablet with 200 mL water;	n.a.	6	-	Caucasian	-	-	-	Suri et al. [39]
Ansaid 100 mg with 180 mL water;	n.a.	15	0	Caucasian	29 (18–40)	76.4 (62.3–109.1)	177 (168–188)	Szpunar et al. [36]
3 × 50 mg conventional tablets (reference);	n.a.	20	0	Chinese	21.4 (2.5)	63.2 (5.1)	174.4 (4.2)	Liu et al. [40]
3 × 50 mg orally disintegrated tablets (test);	n.a.	20	0	Chinese	21.4 (2.5)	63.2 (5.1)	174.4 (4.2)	Liu et al. [40]
2 × Ansaid 100 mg with 180 mL water;	n.a.	15	0	Caucasian	29 (18–40)	76.4 (62.3–109.1)	177 (168–188)	Szpunar et al. [36]
3 × Ansaid 100 mg with 180 mL water;	n.a.	15	0	Caucasian	29 (18–40)	76.4 (62.3–109.1)	177 (168–188)	Szpunaret al. [36]

n.a.: not available.

**Table 2:** Mean (SD) demographic clinical study data used for the gene–drug–drug interaction (GDDI) modeling.

**Victim Drug Administration**	**Perpetrator Drug Administration**	**Perpetrator *in vitro* Ki (μΜ)**	**No. of Doses**	**Interval (h)**	**CYP2C9 genotype**	**No. of Subjects**	**Female Ratio**	**Ethnicity**	**Age (years)**	**BW/BW Range (kg)**	**BH/BH Range (cm)**	**References**
**Flurbiprofen**	**Fluconazole**											
po 50 mg s.d.	po 200 mg q.d.	11	7	2	1*/1*	11	0.64	-	25 (19–36)	73.7 (51–108)	166 (154–193)	Kumar et al. [43]
po 50 mg s.d.	po 400 mg q.d.	11	7	2	1*/1*	11	0.64	-	25 (19–36)	73.7 (51–108)	166 (154–193)	Kumar et al. [43]
po 50 mg s.d.	po 200 mg q.d.	17	7	2	1*/3*	8	0.63	-	23 (19–28)	66.9 (49–84)	167 (160–189)	Kumar et al. [43]
po 50 mg s.d.	po 400 mg q.d.	17	7	2	1*/3*	8	0.63	-	23 (19–28)	66.9 (49–84)	167 (160–189)	Kumar et al. [43]
po 50 mg s.d.	po 200 mg q.d.	23	7	2	3*/3*	2	0.0	-	(25, 29)	(77, 85)	(177, 179)	Kumar et al. [43]
po 50 mg s.d.	po 400 mg q.d.	23	7	2	3*/3*	2	0.0	-	(25, 29)	(77, 85)	(177, 179)	Kumar et al. [43]
po 100 mg s.d.	po 200 mg b.i.d.	14.3/20.3	2	0.5	-	12	0.25	Caucasian (*n* = 8), other (*n* = 4) ^a^	19–54	-	-	Hanley et al. [45]
po 100 mg s.d.	po 200 mg b.i.d.	29.9	2	0.5	-	14	0.21	-	29 ± 8	81 ± 14	-	Greenblatt et al. [46]
po 100 mg s.d.	po 200 mg b.i.d.	14.3/20.3	2	0.5	-	12	0.17	-	24–55	-	-	Hanley et al. [44]
**Victim Drug Administration**	**Perpetrator Drug Administration**	**Perpetrator *in vitro* Ki (μΜ)**	**No. of Doses**	**Interval (h)**	**CYP2C9 genotype**	**No. of Subjects**	**Female Ratio**	**Ethnicity**	**Age (years)**	**BW/BW Range (kg)**	**BH/BH Range (cm)**	**References**
**Flurbiprofen**	**Fluconazole**											
po 50 mg s.d.	po 400 mg s.d	10	1	2	-	12	0.58	Caucasian (*n* = 10), other (*n* = 2) ^b^	37 ± 3.1	-	-	Zgheib et al. [47]
po 50 mg s.d.	po 400 mg q.d	10	7	2	-	12	0.58	Caucasian (*n* = 10), other (*n* = 2) ^b^	37 ± 3.1	-	-	Zgheib et al. [47]
po 50 mg s.d.	po 400 mg s.d	10	1	2	-	10	0.0	Caucasian (*n* = 9), African (*n* = 1)	27 (23–39)	-	-	Daali et al. [48]
	**Rifampicin**											
po 50 mg s.d.	po 600 mg q.d.	n.a.^c^	5	0	-	10	0.0	Caucasian (*n* = 9), African (*n* = 1)	27 (23–39)	-	-	Daali et al. [48]

n.a. = not available; ^a^ Hispanic (*n* = 2), Asian (*n* = 1), Afro-American (*n* = 1); ^b^ Afro-American (*n* = 2); ^c^ default value of Simcyp library compound.

**Table 3:** Input parameters of flurbiprofen PBPK/PD model.

Parameters	Value	Reference/Comments
**Physicochemical and Blood Binding**	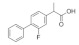	
Chemical Structure		
MW (g/mol)	244.3	
logP_o:w_	3.99	[54,55]
pKa	4.05	Updated from *in vitro* solubility data (see Table 4 and Section 3.2)
Blood/plasma ratio	0.55	[56]
Fraction unbound in plasma	0.01	[5,56,57,58,59]
**Absorption**		
Model	ADAM	
P_app, Caco-2_ (×10^−6^ cm/s)	20.1	Measured value [60]
P_app, Caco-2, ref_ (×10^−6^ cm/s)	1.57	Negative calibrator (Atenolol) value [60]
P_app, Caco-2, ref_ (×10^−6^ cm/s)	15.8	Positive calibrator (Verapamil) value [60]
P_eff, human_ (×10^−4^ cm/s)	4.83	Predicted by Simcyp Permeability Calibrator-custom correlation
Formulation type	Immediate Release	
S_0_ (mg/mL)	0.018	*In vitro* data (see Table 4 and Section 3.1)
logK_m:w_ neutral	5.37	Estimated from *in vitro* data (see Table 5 and Section 2.6 and Section 3.2)
logK_m:w_ ion	2.46	Estimated from *in vitro* data (see Table 5 and Section 2.6 and Section 3.2)
*In vivo* dissolution	see Table 6 and Table 7	Estimated DLM scalars from *in vitro* data (see Section 2.7)
**Distribution**		
Model	Full PBPK	
V_ss_ (L/kg)	0.074	Predicted by Method 2
Kp scalar	0.7	Optimized on the basis of IV data–PE module
**Elimination**		
F_ox_	0.71	[4]
Model	Allelic-specific enzyme kinetics	
CYP2C9 1*/1*-V_max_ (pmol/min/pmol CYP)	15.79	Recombinant CYP (f_u,mic_ = 1) [61]
CYP2C9 1*/1*-K_m_ (μM)	8.756	Recombinant CYP (f_u,mic_ = 1) [61]
CYP2C9 1*/2*-V_max_ (pmol/min/pmol CYP)	11.53	Scaled for CL_CYP2C9 1*/1*_/CL_CYP2C9 1*/3*_ = 0.73 [42]
CYP2C9 1*/2*-K_m_ (μM)	8.756	Recombinant CYP (f_u,mic_ = 1) [61]
CYP2C9 1*/3*-V_max_ (pmol/min/pmol CYP)	9.55	Scaled for CL_CYP2C9 1*/1*_/CL_CYP2C9 1*/3*_ = 0.605 [42]
CYP2C9 1*/3*-K_m_ (μM)	8.756	Recombinant CYP (f_u,mic_ = 1) [61]
CYP2C9 2*/2*-V_max_ (pmol/min/pmol CYP)	10.04	Recombinant CYP (f_u,mic_ = 1) [61]
CYP2C9 2*/2*-K_m_ (μM)	10.39	Recombinant CYP (f_u,mic_ = 1) [61]
CYP2C9 3*/3*-V_max_ (pmol/min/pmol CYP)	8.901	Recombinant CYP (f_u,mic_ = 1) [61]
CYP2C9 3*/3*-K_m_ (μM)	23.25	Recombinant CYP (f_u,mic_ = 1) [61]
CYP2C9-ISEF	0.3	Optimized on the basis of IV data–PE module
UGT2B7-V_max_ (pmol/min/mg protein)	119.7	Recombinant UGT [62]
UGT2B7-K_m_ (μM)	50.21	Recombinant UGT [62]
UGT1A9-V_max_ (pmol/min/mg protein)	3.286	Recombinant UGT [62]
UGT1A9-K_m_ (μM)	182.2	Recombinant UGT [62]
Additional HLM liver CL_int_ (μL/min/mg proein)	7.88	Retrograde model for a target f_mCYP2C9_ = 0.71
Cl_renal_ (L/h)	0.066	[4]
Pharmacodynamics		
Model	Effect compartment linked to inhibitory E_max_ model	[39]
k_eo_ (h^−1^) (%CV)	0.56 (43)	PD endpoint: *Evoked Potentials*
IC_50_ (mg/L)	25.8 (21)	
k_eo_ (h^−1^) (%CV)	0.89 (24)	PD endpoint: *Pain rating score*
IC_50_ (mg/L)	27.6 (10)	

**Table 4:** Mean (± SD) equilibrium solubility in aqueous buffers and fasted state biorelevant media at 37 °C for 24 h (Uniprep method).

Medium	Flurbiprofen	
	pH_final_	Solubility (μg/mL)
Aqueous buffers		
FaSSGF Level I (pH = 1.6)	1.6	18.1 (0.17)
Acetate buffer (pH = 4.5)	4.7	101.1 (7.06)
FeSSIF V1 Level I (pH = 5.0)	5.1	225.4 (5.6)
Phosphate buffer (pH = 6.5)	6.1	2024.4 (128.2)
Phosphate buffer (pH = 6.8)	6.3	3127.1 (194.9)
Fasted state biorelevant media		
Level III FaSSGF (pH = 1.6)	1.6	18.5 (1.6)
Level II FaSSIF V1 (pH = 6.5)	6.0	1954.9 (3.9)
Level II FaSSIF V3 (pH = 6.7)	5.9	1585.4 (172.1)

**Table 5:** Parameter estimates (95% CI) resulting from the model-based analysis of *in vitro* solubility data in aqueous as well as biorelevant media. The pKa was estimated from the aqueous buffer solubility values, whereas for the micelle-water partition coefficients (logK_m:w_ neutral, ion) estimation, we used biorelevant solubilities. The accuracy of the prediction was evaluated with the *R^2^*.

	pKa	logK_m:w_ Neutral	logK_m:w_ Ion
Estimate (95% CI)	4.05 (4.42–4.44)	5.36 (4.61–6.11)	2.56 (1.38–5.02)
*R^2^*	0.9990	0.9999	

**Table 6:** Mean (95% CI) diffusion layer model (DLM) scalar (S_DLM_) estimates obtained from model-based analysis of *in vitro* dissolution data in various media for flurbiprofen pure drug, 100 mg USP tablets, and 100 mg Antadys formulations. The goodness of fit between predicted and observed dissolution profiles was evaluated with the *R^2^*.

Dissolution Medium	Formulation		
	API Powder	USP Tablets	Antadys
**FaSSGF Level III**			
S_DLM_ (95% CI)	0.0218 (0.0161–0.0274)	0.0929 (0.0731–0.113)	0.107 (0.087–0.127)
*R^2^*	0.944	0.973	0.982
**FaSSIF V3 Level I**			
S_DLM_ (95% CI)	0.00185 (0.001–0.00312)	0.0791 (0.0589–0.993)	0.120 (0.0979–0.142)
*R^2^*	0.974	0.986	0.995
**FaSSIF V3 Level II**			
S_DLM_ (95% CI)	0.0965 (0.0544–0.139)	0.0622 (0.0398–0.0847)	0.125 (0.106–0.143)
*R^2^*	0.971	0.976	0.996
**Ph. Eur. Phosphate Buffer**			
S_DLM_ (95% CI)	0.00542 (0.00468–0.00617)	0.0150 (0.0110–0.0189)	0.0449 (0.0448–0.0450)
*R^2^*	0.986	0.983	0.999

**Table 7:** Mean (95% CI) DLM scalar (S_DLM_) estimates obtained from model-based analysis of Froben *in vitro* dissolution data. The *in vitro* data from single dissolution experiments were modelled under the assumption that disintegration is the rate-limiting step to flurbiprofen dissolution in intestinal media, whereas for the two-stage dissolution, the serial dilution model was used. The goodness of fit between predicted and observed dissolution profiles was evaluated with the *R^2^*.

Dissolution Model/Media	Formulation
	Froben
**First order disintegration/all intestinal media**	
kd (h^−1^) (95% CI)	0.127 (0.00844–0.0253)
Tlag (min) (95% CI)	14.6 (8.91–20.1)
*R^2^*	0.941
**Serial Dilution/Two-stage (FaSSGF Level III + FaSSIF V3 Level II)**	
S_DLM, Gastric_ (95% CI)	0.001 (0.001–0.0244)
S_DLM, Intestinal_ (95% CI)	0.0712 (0.0576–0.0849)
*R^2^*	0.991

**Table 8:** Mean relative deviation (MRD) values of flurbiprofen plasma concentration predictions.

Route of Administration	Dose (mg)	Flurbiprofen MRD	Reference
***iv (s.d.)***	50	1.41	[34]
***po (sol, s.d.)***	67.9	1.85	[35]
***po (sol, s.d.)***	100	1.60	[36]
po (sol, s.d., CYP2C9 1*/1*)	40	1.52	[37]
po (sol, s.d., CYP2C9 1*/3*)	40	1.30	[37]
po (tab, s.d., CYP2C9 1*/1*)	50	1.24	[42]
po (tab, s.d., CYP2C9 1*/2*)	50	1.30	[42]
po (tab, s.d., CYP2C9 1*/3*)	50	1.62	[42]
po (tab, s.d., CYP2C9 1*/1*)	50	1.26	[43]
po (tab, s.d., CYP2C9 1*/1*)	50	1.25	[43]
po (tab, s.d., CYP2C9 1*/3*)	50	1.28	[43]
po (tab, s.d., CYP2C9 1*/3*)	50	1.25	[43]
po (tab, s.d., CYP2C9 3*/3*)	50	1.25	[43]
po (tab, s.d., CYP2C9 3*/3*)	50	1.20	[43]
***po (tab (Ansaid), s.d.)***	100	1.31–2.38	[38]
*S_DLM_SI_ = 0.125*		1.31	
*S_DLM_SI_ = 0.0054*		1.72	
*k_d_ = 0.127 h^−1^ and T_lag_ = 14.6 min*		1.64	
*S_DLM_SI_ = 0.0712*		1.65	
*S_DLM_SI_ = 0.0018*		2.38	
***po (tab (Froben), s.d.)***	100	1.70–2.43	[38]
*S_DLM_SI_ = 0.125*		1.70	
*S_DLM_SI_ = 0.0054*		1.88	
*k_d_ = 0.127 h^−1^ and T_lag_ = 14.6 min*		1.80	
*S_DLM_SI_ = 0.0712*		1.83	
*S_DLM_SI_ = 0.0018*		2.43	
***po (tab (Froben), s.d.)***	100	1.69–1.92	[4]
*S_DLM_SI_ = 0.125*		1.69	
*S_DLM_SI_ = 0.0054*		1.78	
*k_d_ = 0.127 h^−1^ and T_lag_ = 14.6 min*		1.92	
*S_DLM_SI_ = 0.0712*		1.90	
*S_DLM_SI_ = 0.0018*		1.79	
***po (tab, s.d.)***	100	1.04–1.74	[39]
*S_DLM_SI_ = 0.125*		1.11	
*S_DLM_SI_ = 0.0054*		1.20	
*k_d_ = 0.127 h^−1^ and T_lag_ = 14.6 min*		1.12	
*S_DLM_SI_ = 0.0712*		1.04	
*S_DLM_SI_ = 0.0018*		1.74	
***po (tab, s.d)***	150	1.51	[40]
***po (orod, s.d.)***	150	1.42	[40]
***po (tab (Ansaid), s.d.)***	200	1.20	[36]
***po (tab (Ansaid), s.d.)***	300	1.34	[36]
***MRD (range)***		1.54 (1.04–2.43)	
***MRD*** ***≤ 1.25***		*9/38*	
***MRD*** ***≤ 2***		*36/38*	

**Table 9:** Comparison of mean predicted and observed AUC, C_max_, and apparent clearance (CL/F) values of flurbiprofen. Calculation of predicted to observed ratio (R_pred/obs_) and geometric fold error (GMFE) values.

		AUC_inf_ (mg/L·h)			C_max_ (mg/L)			CL/F (L/h)			
Route of Administration	Dose (mg)	obs	pred	R_pred/obs_	obs	pred	R_pred/obs_	obs	pred	R_pred/obs_	Reference
**iv (s.d.)**	50	35.2	43.7	1.24	-	-	-	1.50	1.36	0.91	[34]
**po (sol, s.d.)**	67.9	55.1	56.1	1.01	10.8	9.99	0.92	*-*	*-*	*-*	[35]
**po (sol, s.d.)**	100	82.7	78.0	0.94	14.2	12.9	0.91	1.28	1.50	1.17	[36]
po (sol, s.d., CYP2C9 1*/1*)	40	29.3	29.1	0.99	5.54	5.86	1.06	1.39	1.16	0.83	[37]
po (sol, s.d., CYP2C9 1*/3*)	40	47.6	44.2	0.93	6.93	6.22	0.90	0.88	0.67	0.76	[37]
po (tab, s.d., CYP2C9 1*/1*)	50	29.4	28.6	0.97	5.38	5.84	1.09	1.77	1.67	0.83	[42]
po (tab, s.d., CYP2C9 1*/2*)	50	40.7	45.6	1.12	4.55	6.34	1.39	1.30	1.20	0.92	[42]
po (tab, s.d., CYP2C9 1*/3*)	50	51.1	46.4	0.91	5.42	6.68	1.23	1.00	1.03	1.03	[42]
po (tab, s.d., CYP2C9 1*/1*)	50	30.8^a^	35.8 ^a^	1.16	6.1 ^a^	6.91 ^a^	1.13	1.6 ^a^	1.4 ^a^	0.88	[43]
po (tab, s.d., CYP2C9 1*/1*)	50	30.8 ^a^	36 ^a^	1.17	6.1 ^a^	6.8 ^a^	1.11	1.6 ^a^	1.43 ^a^	0.89	[43]
po (tab, s.d., CYP2C9 1*/3*)	50	53.7 ^a^	54.6 ^a^	1.02	8.9 ^a^	7.7 ^a^	0.87	0.9 ^a^	0.98 ^a^	1.09	[43]
po (tab, s.d., CYP2C9 1*/3*)	50	53.7 ^a^	53.1 ^a^	0.99	8.9 ^a^	7.44 ^a^	0.84	0.9 ^a^	0.96 ^a^	1.07	[43]
po (tab, s.d., CYP2C9 3*/3*)	50	(85.8, 119) ^b^	76.1	(0.89, 0.64)	(8, 9.4) ^b^	6.99	(0.87, 0.74)	(0.6, 0.4) ^b^	0.64	(1.07, 1.6)	[43]
po (tab, s.d., CYP2C9 3*/3*)	50	(85.8, 119) ^b^	77.7	(0.91, 0.65)	(8, 9.4) ^b^	7.1	(0.89, 0.76)	(0.6, 0.4) ^b^	0.68	(1.13, 1.7)	[43]
**po (tab (Ansaid), s.d.)**	100										[38]
S_DLM_SI_ = 0.125		80.5	81.1	1.01	12.8	12.8	1.00	*-*	*-*	*-*	
S_DLM_SI_ = 0.0054			66.5	0.83		11.6	0.90				
k_d_ = 0.127 h^−1^ and T_lag_ = 14.6 min			68.8	0.85		11.8	0.92				
S_DLM_SI_ = 0.0712			67.2	0.83		12.3	0.96				
S_DLM_SI_ = 0.0018			62.7	0.78		10.4	0.81				
**po (tab (Froben), s.d.)**	100										[38]
S_DLM_SI_ = 0.125		82.3	81.1	0.99	13.3	12.8	0.96	*-*	*-*	*-*	
S_DLM_SI_ = 0.0054			66.5	0.81		11.6	0.87				
k_d_ = 0.127 h^−1^ and T_lag_ = 14.6 min			68.8	0.84		11.8	0.88				
S_DLM_SI_ = 0.0712			67.2	0.82		12.3	0.92				
S_DLM_SI_ = 0.0018			62.7	0.76		10.4	0.78				
**po (tab (Froben), s.d.)**	100										[4]
S_DLM_SI_ = 0.125		87.8	81.1	0.92	13.2	12.7	0.96	1.27	1.42	1.12	
S_DLM_SI_ = 0.0054			66.5	0.76		11.6	0.88		1.68	1.32	
*k_d_ = 0.127 h^−1^ and T_lag_ = 14.6 min*			68.8	0.78		11.8	0.89		1.66	1.31	
*S_DLM_SI_ = 0.0712*			67.2	0.77		12.2	0.92		1.66	1.31	
*S_DLM_SI_ = 0.0018*			62.7	0.71		11.0	0.84		1.77	1.39	
*po (tab, s.d.)*	100										[39]
*S_DLM_SI_ = 0.125*		67.7	81.1	1.20	12.9	12.7	0.98	1.52	1.42	0.93	
*S_DLM_SI_ = 0.0054*			66.5	0.98		11.6	0.90		1.68	1.11	
*k_d_ = 0.127 h^−1^ and T_lag_ = 14.6 min*			68.8	1.02		11.8	0.91		1.66	1.09	
*S_DLM_SI_ = 0.0712*			67.2	0.99		12.2	0.95		1.66	1.09	
*S_DLM_SI_ = 0.0018*			62.7	0.93		11.0	0.86		1.77	1.16	
***po (tab, s.d)***	150	124.3	154.4	1.24	15.2	20.7	1.36	*-*	*-*	*-*	[40]
***po (orod, s.d.)***	150	129.8	154.4	1.19	16.8	20.7	1.23	*-*	*-*	*-*	[40]
***po (tab (Ansaid), s.d.)***	200	161.3	159.9	0.99	21.4	23.6	1.10	1.32	1.50	1.14	[36]
***po (tab (Ansaid), s.d.)***	300	233.9	228.9	0.98	29.5	33.7	1.14	1.36	1.55	1.14	[36]
***GMFE (range)***		1.15 (1.01–1,56)			1.14 (1.00–1.39)			1.18 (1.03–1.7)			
***GMFE*** ***≤ 1.25***		*30/38*			*31/37*			*18/25*			
***GMFE*** ***≤ 2***		*38/38*			*37/37*			*25/25*			

^a^: median value; ^b^: individual values (*n* = 2).

**Table 10:** Comparison of mean predicted and observed drug–drug interaction (DDI) AUC, C_max_, and apparent clearance (CL/F) ratios of flurbiprofen–fluconazole/rifampicin interaction. Calculation of predicted to observed ratio (R_pred/obs_) and geometric fold error (GMFE) values.

					*DDI AUC Ratio*			*DDI C_max_ Ratio*			*DDI CL/F Ratio*			Reference
Victim Drug Administration	Perpetrator Drug Administration	No. of Doses	Interval (h)	CYP2C9 Genotype	obs	pred	R_pred/obs_	obs	pred	R_pred/obs_	obs	pred	R_pred/obs_	
**Flurbiprofen**	**Fluconazole**													
po 50 mg s.d.	po 200 mg q.d.	7	2	1*/1*	2.02 ^a^	1.94	0.96	1.03 ^a^	1.18	1.15	0.5 ^a^	0.51	1.02	[43]
po 50 mg s.d.	po 400 mg q.d.	7	2	1*/1*	3.03	2.36	0.78	0.99	1.23	1.24	0.31	0.42	1.35	[43]
po 50 mg s.d.	po 200 mg q.d.	7	2	1*/3*	1.8	1.58	0.88	0.87	1.11	1.28	0.56	0.63	1.13	[43]
po 50 mg s.d.	po 400 mg q.d.	7	2	1*/3*	2.48	1.84	0.74	0.94	1.14	1.21	0.44	0.54	1.23	[43]
po 50 mg s.d.	po 200 mg q.d.	7	2	3*/3*	(1.58, 1.28)^#^	1.09	0.76	(1.08, 0.91) ^#^	1.02	1.02	(0.75, 0.66) ^#^	0.92	1.30	[43]
po 50 mg s.d.	po 400 mg q.d.	7	2	3*/3*	(1.39, 1.12)^#^	1.16	0.92	(0.54, 0.90) ^#^	1.03	1.43	(1.00, 0.66) ^#^	0.86	1.04	[43]
po 100 mg s.d.	po 200 mg b.i.d.	2	0.5	n.a.	1.71 ^b^	1.65	0.97	1.16 ^b^	1.15	0.99	0.57	0.61	1.07	[45]
po 100 mg s.d.	po 200 mg b.i.d.	2	0.5	n.a.	1.81	1.51	0.83	1.23	1.13	0.92	0.55	0.68	1.24	[46]
po 100 mg s.d.	po 200 mg b.i.d.	2	0.5	n.a.	1.97 ^b^	1.62	0.82	1.47 ^b^	1.15	0.78	0.5	0.62	1.24	[44]
po 50 mg s.d.	po 400 mg s.d.	1	2	n.a.	2.16	2.23	1.03	1.24	1.2	0.97	0.46	0.48	1.04	[47]
po 50 mg s.d.	po 400 mg q.d.	7	2	n.a.	2.81	2.87	1.02	1.37	1.25	0.91	0.35	0.39	1.11	[47]
po 50 mg s.d.	po 400 mg s.d.	1	2	n.a.	1.21	1.53	1.26	1.14	1.14	1.00	0.67	0.67	1.00	[48]
	**Rifampicin**													
po 50 mg s.d.	po 600 mg q.d.	5	0	n.a.	0.56	0.63	1.13	0.71	0.83	1.17	1.85	1.73	0.94	Daali et al.
**GMFE (range)**					1.17 (1.02–1.35)			1.16 (1.00–1.43)			1.15 (1.00–1.35)			
**GMFE** **≤ 1.25**					8/12			9/12			10/12			
**GMFE** **≤ 2**					12/12			12/12			12/12			

n.a.= not available; ^a^ median; ^b^ geometric mean. ^#^ individual values (n=2)

## Supplementary Materials

The following are available online at https://www.mdpi.com/1999-4923/12/11/1049/s1, Table S1: Summary of main CYP2C9 genotype-based metabolic differences in the default inputs of the Simcyp North European Caucasian (NEurCaucasian) and Chinese healthy volunteer virtual populations. Table S2: Comparison of predicted and observed pharmacodynamic parameters (R_max_, TR_max_, and AUCE) values of flurbiprofen and calculation of predicted to observed ratio (R_pred/obs_).

## References

[1] Davies N.M. (1995). Clinical pharmacokinetics of flurbiprofen and its enantiomers. Clin. Pharm..

[2] Dressman J.B., Berardi R.R., Elta G.H., Gray T.M., Montgomery P.A., Lau H.S., Pelekoudas K.L., Szpunar G.J., Wagner J.G. (1992). Absorption of flurbiprofen in the fed and fasted states. Pharm. Res..

[3] Ozbay L., Unal D.O., Cakici I., Fenercioglu A., Erol D. (2009). Clinical study on the bioequivalence of two tablet formulations of flurbiprofen. Eur. J. Drug Metab. Pharm..

[4] Patel B.K., Jackson S.H.D., Swift C.G., Hutt A.J., Jackson S.H.D., Swift C.G., Disposition A.J.H. (2003). Disposition of flurbiprofen in man: Influence of stereochemistry and age. Xenobiotica.

[5] Szpunar G.J., Albert K.S., Wagner J.G. (1989). Pharmacokinetics of flurbiprofen in man. II. Plasma protein binding. Res. Commun. Chem. Pathol. Pharmacol..

[6] Human Cytochrome P450 (CYP) Allele Nomenclature Committee CYP2C9 Allele Nomenclature. https://www.pharmvar.org/htdocs/archive/cyp2c9.htm.

[7] Yamazaki H., Inoue K., Chiba K., Ozawa N., Kawai T., Suzuki Y., Goldstein J.A., Guengerich F.P., Shimada T. (1998). Comparative Studies on the Catalytic Roles of Cytochrome P450 2C9 and Its Cys-and Leu-Variants in the Oxidation of Warfarin, Flurbiprofen, and Diclofenac by Human Liver Microsomes. Biochem. Pharmacol..

[8] Van Booven D., Marsh S., McLeod H., Carrillo M.W., Sangkuhl K., Klein T.E., Altman R.B. (2010). Cytochrome P450 2C9-CYP2C9. Pharmacogenet. Genom..

[9] Scott S.A., Khasawneh R., Peter I., Kornreich R., Desnick R.J. (2010). Combined CYP2C9, VKORC1 and CYP4F2 frequencies among racial and ethnic groups. Pharmacogenomics.

[10] Kirchheiner J., Brockmöller J. (2005). Clinical consequences of cytochrome P450 2C9 polymorphisms. Clin. Pharmacol. Ther..

[11] Lee C.R., Goldstein J.A., Pieper J.A. (2002). Cytochrome P450 2C9 polymorphisms: A comprehensive review of the in-vitro and human data. Pharmacogenetics.

[12] Vieira M.D.L.T., Kim M.J., Apparaju S., Sinha V., Zineh I., Huang S.M., Zhao P. (2014). PBPK model describes the effects of comedication and genetic polymorphism on systemic exposure of drugs that undergo multiple clearance pathways. Clin. Pharmacol. Ther..

[13] Jin Y., Borell H., Gardin A., Ufer M., Huth F., Camenisch G. (2018). In vitro studies and in silico predictions of fluconazole and CYP2C9 genetic polymorphism impact on siponimod metabolism and pharmacokinetics. Eur. J. Clin. Pharmacol..

[14] Abend A., Heimbach T., Cohen M., Kesisoglou F., Pepin X., Suarez-Sharp S. (2018). Dissolution and Translational Modeling Strategies Enabling Patient-Centric Drug Product Development: The M-CERSI Workshop Summary Report. AAPS J..

[15] Sager J.E., Yu J., Ragueneau-Majlessi I., Isoherranen N. (2015). Minireview Physiologically Based Pharmacokinetic (PBPK) Modeling and Simulation Approaches: A Systematic Review of Published Models, Applications, and Model Verifications. DRUG Metab. Dispos. Drug Metab. Dispos..

[16] Zhao P., Rowland M., Huang S.-M. (2012). Best practice in the use of physiologically based pharmacokinetic modeling and simulation to address clinical pharmacology regulatory questions. Clin. Pharmacol. Ther..

[17] Djebli N., Fabre D., Boulenc X., Fabre G., Sultan E., Hurbin F. (2015). Physiologically Based Pharmacokinetic Modeling for Sequential Metabolism: Effect of CYP2C19 Genetic Polymorphism on Clopidogrel and Clopidogrel Active Metabolite Pharmacokinetics. DRUG Metab. Dispos. Drug Metab. Dispos..

[18] Storelli F., Desmeules J., Daali Y. (2019). Physiologically-Based Pharmacokinetic Modeling for the Prediction of CYP2D6-Mediated Gene–Drug–Drug Interactions. CPT Pharmacomet. Syst. Pharmacol..

[19] Chen Y., Liu L., Nguyen K., Fretland A.J. (2011). Utility of Intersystem Extrapolation Factors in Early Reaction Phenotyping and the Quantitative Extrapolation of Human Liver Microsomal Intrinsic Clearance Using Recombinant Cytochromes P450. Drug Metab. Dispos..

[20] Alqahtani S., Kaddoumi A. (2015). Development of physiologically based pharmacokinetic/Pharmacodynamic model for Indomethacin disposition in pregnancy. PLoS ONE.

[21] Riedmaier A.E., Lindley D.J., Hall J.A., Castleberry S., Slade R.T., Stuart P., Carr R.A., Borchardt T.B., Bow D.A.J., Nijsen M. (2018). Mechanistic Physiologically Based Pharmacokinetic Modeling of the Dissolution and Food Effect of a Biopharmaceutics Classification System IV Compound-The Venetoclax Story. J. Pharm. Sci..

[22] Türk D., Hanke N., Wolf S., Frechen S., Eissing T., Wendl T., Schwab M., Lehr T. (2019). Physiologically Based Pharmacokinetic Models for Prediction of Complex CYP2C8 and OATP1B1 (SLCO1B1) Drug-Drug-Gene Interactions: A Modeling Network of Gemfibrozil, Repaglinide, Pioglitazone, Rifampicin, Clarithromycin and Itraconazole. Clin. Pharm..

[23] Markopoulos C., Andreas C.J., Vertzoni M., Dressman J., Reppas C. (2015). In-vitro simulation of luminal conditions for evaluation of performance of oral drug products: Choosing the appropriate test media. Eur. J. Pharm. Biopharm..

[24] Fuchs A., Leigh M., Kloefer B., Dressman J.B. (2015). Advances in the design of fasted state simulating intestinal fluids: FaSSIF-V3. Eur. J. Pharm. Biopharm..

[25] Loisios-Konstantinidis I., Cristofoletti R., Fotaki N., Turner D.B., Dressman J. (2020). Establishing virtual bioequivalence and clinically relevant specifications using in vitro biorelevant dissolution testing and physiologically-based population pharmacokinetic modeling. case example: Naproxen. Eur. J. Pharm. Sci..

[26] Wang J., Flanagan D.R. (1999). General solution for diffusion-controlled dissolution of spherical particles. 1. Theory. J. Pharm. Sci..

[27] Wang J., Flanagan D.R. (2002). General solution for diffusion-controlled dissolution of spherical particles. 2. Evaluation of experimental data. J. Pharm. Sci..

[28] Mooney K.G., Mintun M.A., Himmelstein K.J., Stella V.J. (1981). Dissolution kinetics of carboxylic acids I: Effect of pH under unbuffered conditions. J. Pharm. Sci..

[29] Mooney K.G., Mintun M.A., Himmelstein K.J., Stella V.J. (1981). Dissolution Kinetics of Carboxylic Acids II: Effect of Buffers. J. Pharm. Sci..

[30] Mooney K.G., Rodriguez-gaxiola M., Mintun M., Himmelstein K.J., Stella V.J. (1981). Dissolution Kinetics of Phenylbutazone. J. Pharm. Sci..

[31] Ozturk S.S., Palsson B.O., Dressman J.B. (1988). Dissolution of lonizable Drugs in Buffered and Unbuffered Solutions. Pharm. Res..

[32] Sheng J.J., McNamara D.P., Amidon G.L. (2009). Toward an In Vivo dissolution methodology: A comparison of phosphate and bicarbonate buffers. Mol. Pharm..

[33] Serajuddin A.T.M., Jarowski C. (1985). Effect of diffusion layer pH and solubility on the dissolution rate of pharmaceutical bases and their hydrochloride salts. I: Phenazopyridine. J. Pharm. Sci..

[34] Mei C., Li B., Yin Q., Jin J., Xiong T., He W., Gao X., Xu R., Zhou P., Zheng H. (2015). Liquid chromatography-tandem mass spectrometry for the quantification of flurbiprofen in human plasma and its application in a study of bioequivalence. J. Chromatogr. B Anal. Technol. Biomed. Life Sci..

[35] Gonzalez-Younes I., Wagner J.G., Gaines D.A., Ferry J.J., Hageman J.M. (1991). Absorption of flurbiprofen through human buccal mucosa. J. Pharm. Sci..

[36] Szpunar G.J., Albert K.S., Bole G.G., Dreyfus J.N., Lockwood G.F., Wagner J.G. (1987). Pharmacokinetics of flurbiprofen in man. I. Area/dose relationships. Biopharm. Drug Dispos..

[37] Lee Y.J., Byeon J.Y., Kim Y.H., Kim S.H., Choi C.I., Bae J.W., Sohn U.D., Jang C.G., Lee J., Lee S.Y. (2015). Effects of CYP2C9∗1/∗3 genotype on the pharmacokinetics of flurbiprofen in Korean subjects. Arch. Pharm. Res..

[38] Jamali F., Collins D.S., Berry B.W., Molder S., Cheung R., McColl K., Cheung H. (1991). Comparative bioavailability of two flurbiprofen products: Stereospecific versus conventional approach. Biopharm. Drug Dispos..

[39] Suri A., Grundy B.L., Derendorf H. (1997). Pharmacokinetics and pharmacodynamics of enantiomers of ibuprofen and flurbiprofen after oral administration. Int. J. Clin. Pharmacol. Ther..

[40] Liu Y.-M., Liu G.-Y., Liu Y., Li S.-J., Jia J.-Y., Zhang M.-Q., Lu C., Zhang Y.-M., Li X.-N., Yu C. (2009). Pharmacokinetic and Bioequivalence Comparison Between Orally Disintegrating and Conventional Tablet Formulations of Flurbiprofen: A Single-Dose, Randomized-Sequence, Open-Label, Two-Period Crossover Study in Healthy Chinese Male Volunteers. Clin. Ther..

[41] Lee H.-I., Choi C.-I., Byeon J.-Y., Lee J.-E., Park S.-Y., Kim Y.-H., Kim S.-H., Lee Y.-J., Jang C.-G., Lee S.-Y. (2014). Simultaneous determination of flurbiprofen and its hydroxy metabolite in human plasma by liquid chromatography-tandem mass spectrometry for clinical application. J. Chromatogr. B.

[42] Lee C.R., Pieper J.A., Frye R.F., Hinderliter A.L., Blaisdell J.A., Goldstein J.A. (2003). Differences in flurbiprofen pharmacokinetics between CYP2C9*1/*1, *1/*2, and *1/*3 genotypes. Eur. J. Clin. Pharmacol..

[43] Kumar V., Brundage R., Oetting W.S., Leppik I.E., Tracy T.S. (2008). Differential Genotype Dependent Inhibition of CYP2C9 in Humans. Drug Metab. Dispos..

[44] Hanley M.J., Masse G., Harmatz J.S., Court M.H., Greenblatt D.J. (2012). Pomegranate juice and pomegranate extract do not impair oral clearance of flurbiprofen in human volunteers: Divergence from in vitro results. Clin. Pharmacol. Ther..

[45] Hanley M.J., Masse G., Harmatz J.S., Cancalon P.F., Dolnikowski G.G., Court M.H., Greenblatt D.J. (2013). Effect of blueberry juice on clearance of buspirone and flurbiprofen in human volunteers. Br. J. Clin. Pharmacol..

[46] Greenblatt D.J., Von Moltke L.L., Perloff E.S., Luo Y., Harmatz J.S., Boston M.A.Z. (2006). Interaction of flurbiprofen with cranberry juice, grape juice, tea, and fluconazole: In vitro and clinical studies. Clin. Pharmacol. Ther..

[47] Zgheib N.K., Frye R.F., Tracy T.S., Romkes M., Branch R.A. (2007). Evaluation of flurbiprofen urinary ratios as in vivo indices for CYP2C9 activity. Br. J. Clin. Pharmacol..

[48] Daali Y., Samer C., Déglon J., Thomas A., Chabert J., Rebsamen M., Staub C., Dayer P., Desmeules J. (2012). Oral flurbiprofen metabolic ratio assessment using a single-point dried blood spot. Clin. Pharmacol. Ther..

[49] U.S. Food and Drug Administration, A Center for Drug Evaluation and Research (CDER) (2018). Physiologically Based Pharmacokinetic Analyses—Format and Content Guidance for Industry.

[50] Kuepfer L., Niederalt C., Wendl T., Schlender J.-F., Willmann S., Lippert J., Block M., Eissing T., Teutonico D. (2016). Applied Concepts in PBPK Modeling: How to Build a PBPK/PD Model. CPT Pharmacomet. Syst. Pharmacol..

[51] Ke A., Barter Z., Rowland-Yeo K., Almond L. (2016). Towards a Best Practice Approach in PBPK Modeling: Case Example of Developing a Unified Efavirenz Model Accounting for Induction of CYPs 3A4 and 2B6. CPT Pharmacomet. Syst. Pharmacol..

[52] European Medicines Agency (EMA) (2018). Committee for Medicinal Products for Human Use (CHMP) Guideline on the Reporting of Physiologically Based Pharmacokinetic (PBPK) Modelling and Simulation.

[53] Shebley M., Sandhu P., Emami Riedmaier A., Jamei M., Narayanan R., Patel A., Peters S.A., Reddy V.P., Zheng M., de Zwart L. (2018). Physiologically Based Pharmacokinetic Model Qualification and Reporting Procedures for Regulatory Submissions: A Consortium Perspective. Clin. Pharmacol. Ther..

[54] Avdeef A. (1998). pH-metric Solubility. 1. Solubility-pH Plots. Gibbs Buffer and pK, in Profiles from Bjerrum the Solid State. Pharm. Pharmacol. Commun. Pharm. Pharmacol. Commun..

[55] Czyrski A. (2019). Determination of the Lipophilicity of Ibuprofen, Naproxen, Ketoprofen, and Flurbiprofen with Thin-Layer Chromatography. J. Chem..

[56] Kaiser D.G., Brooks C.D., Lomen P.L. (1986). Pharmacokinetics of Flurbiprofen. Am. J. Med..

[57] Risdall P.C., Adams S.S., Crampton E.L., Marchant B. (1978). The Disposition and Metabolism of Flurbiprofen in Several Species Including Man. XENOBIOTICA.

[58] Aarons L., Khan A.Z., Grennan D.M., Alam-Siddiqi M. (1985). The binding of flurbiprofen to plasma proteins. J. Pharm. Pharmacol..

[59] Lin J.H., Cocchetto D.M., Duggan D.E. (1987). Protein-binding as a primary determinant of the clinical pharmacokinetic properties of nonsteroidal antiinflammatory drugs. Clin. Pharm..

[60] Yazdanian M., Briggs K., Jankovsky C., Hawi A. (2004). The “High Solubility” Definition of the Current FDA Guidance on Biopharmaceutical Classification System May Be Too Strict for Acidic Drugs. Pharm. Res..

[61] Wang L., Bao S.-H., Pan P.-P., Xia M., Chen M.-C., Liang B.-Q., Dai D.-P., Cai J.-P., Hu G.-X., Xia M.-M. (2015). Effect of CYP2C9 genetic polymorphism on the metabolism of flurbiprofen in vitro. Drug Dev. Ind. Pharm..

[62] Wang H., Yuan L., Zeng S. (2011). Characterizing the effect of UDP-glucuronosyltransferase (UGT) 2B7 and UGT1A9 genetic polymorphisms on enantioselective glucuronidation of flurbiprofen. Biochem. Pharmacol..

[63] Jamei M., Turner D., Yang J., Neuhoff S., Polak S., Rostami-Hodjegan A., Tucker G. (2009). Population-based mechanistic prediction of oral drug absorption. AAPS J..

[64] Darwich A.S., Neuhoff S., Jamei M., Rostami-Hodjegan A. (2010). Interplay of Metabolism and Transport in Determining Oral Drug Absorption and Gut Wall Metabolism: A Simulation Assessment Using the “Advanced Dissolution, Absorption, Metabolism (ADAM)” Model. Curr. Drug Metab..

[65] Hens B., Brouwers J., Anneveld B., Corsetti M., Symillides M., Vertzoni M., Reppas C., Turner D.B., Augustijns P. (2014). Gastrointestinal transfer: In vivo evaluation and implementation in in vitro and in silico predictive tools. Eur. J. Pharm. Sci..

[66] Psachoulias D., Vertzoni M., Goumas K., Kalioras V., Beato S., Butler J., Reppas C. (2011). Precipitation in and Supersaturation of Contents of the Upper Small Intestine After Administration of Two Weak Bases to Fasted Adults. Pharm. Res..

[67] Cristofoletti R., Patel N., Dressman J.B. (2016). Differences in Food Effects for 2 Weak Bases With Similar BCS Drug-Related Properties: What Is Happening in the Intestinal Lumen?. J. Pharm. Sci..

[68] Paixão P., Bermejo M., Hens B., Tsume Y., Dickens J., Shedden K., Salehi N., Koenigsknecht M.J., Baker J.R., Hasler W.L. (2018). Gastric emptying and intestinal appearance of nonabsorbable drugs phenol red and paromomycin in human subjects: A multi-compartment stomach approach. Eur. J. Pharm. Biopharm..

[69] Rodgers T., Rowland M. (2006). Physiologically based pharmacokinetic modelling 2: Predicting the tissue distribution of acids, very weak bases, neutrals and zwitterions. J. Pharm. Sci..

[70] Crewe H.K., Barter Z.E., Rowland Yeo K., Rostami-Hodjegan A. (2011). Are there differences in the catalytic activity per unit enzyme of recombinantly expressed and human liver microsomal cytochrome P450 2C9? A systematic investigation into inter-system extrapolation factors. Biopharm. Drug Dispos..

[71] Kuehl G.E., Lampe J.W., Potter J.D., Bigler J. (2005). Glucuronidation of nonsteroidal intiinflammatory drugs Identifying the enzymes in human liver microsomes. Drug Metab. Dispos..

[72] Mano Y., Usui T., Kamimura H. (2007). Predominant contribution of UDP-glucuronosyltransferase 2B7 in the glucuronidation of racemic flurbiprofen in the human liver. Drug Metab. Dispos..

[73] Nielsen L.M., Sverrisdóttir E., Stage T.B., Feddersen S., Brøsen K., Christrup L.L., Drewes A.M., Olesen A.E. (2017). Lack of genetic association between OCT1, ABCB1, and UGT2B7 variants and morphine pharmacokinetics. Eur. J. Pharm. Sci..

[74] Ayuso P., Neary M., Chiong J., Owen A. (2019). Meta-analysis of the effect of CYP2B6, CYP2A6, UGT2B7 and CAR polymorphisms on efavirenz plasma concentrations. J. Antimicrob. Chemother..

[75] Edginton A.N., Schmitt W., Willmann S. (2006). Development and evaluation of a generic physiologically based pharmacokinetic model for children. Clin. Pharm..

[76] Poulin P., Theil F.-P. (2009). Development of a novel method for predicting human volume of distribution at steady-state of basic drugs and comparative assessment with existing methods. J. Pharm. Sci..

[77] Obach R.S., Baxter J.G., Liston T.E., Silber B.M., Jones B.C., MacIntyre F., Rance D.J., Wastall P. (1997). The prediction of human pharmacokinetic parameters from preclinical and in vitro metabolism data. J. Pharmacol. Exp. Ther..

[78] Kim Y., Hatley O., Rhee S.J., Yi S., Lee H.A., Yoon S., Chung J.Y., Yu K.S., Lee H. (2019). Development of a Korean-specific virtual population for physiologically based pharmacokinetic modelling and simulation. Biopharm. Drug Dispos..

[79] Myrand S.P., Sekiguchi K., Man M.Z., Lin X., Tzeng R.Y., Teng C.H., Hee B., Garrett M., Kikkawa H., Lin C.Y. (2008). Pharmacokinetics/genotype associations for major cytochrome P450 enzymes in native and first- and third-generation Japanese populations: Comparison with Korean, Chinese, and Caucasian populations. Clin. Pharmacol. Ther..

[80] Yoon Y.-R., Shon J.-H., Kim M.-K., Lim Y.-C., Lee H.-R., Park J.-Y., Cha I.-J., Shin J.-G. (2008). Frequency of cytochrome P450 2C9 mutant alleles in a Korean population. Br. J. Clin. Pharmacol..

[81] Avdeef A., Berger C.M. (2000). pH-Metric Solubility. 2: Correlation Between the Acid-Base Titration and formulations for use in early animal bioavailability and toxicity studies. Later in development, solubility takes on a broader. Pharm. Res..

[82] Karow A.R., Bahrenburg S., Garidel P. (2013). Buffer capacity of biologics-from buffer salts to buffering by antibodies. Biotechnol. Prog..

[83] Lee C.B., Pieper J.A., Frye R.F., Hinderliter A.L., Blaisdell J.A., Goldstein J.A. (2003). Tolbutamide, flurbiprofen, and losartan as probes of CYP2C9 activity in humans. J. Clin. Pharmacol..

[84] Vogl S., Lutz R.W., Schönfelder G., Lutz W.K. (2015). CYP2C9 genotype vs. metabolic phenotype for individual drug dosing-A correlation analysis using flurbiprofen as probe drug. PLoS ONE.

[85] EMA Committee for Proprietary Medicinal Products (CPMP) (2000). Note for Guidance on the Investigation of Bioavailability and Bioequivalence.

[86] Dai D.P., Xu R.A., Hu L.M., Wang S.H., Geng P.W., Yang J.F., Yang L.P., Qian J.C., Wang Z.S., Zhu G.H. (2014). CYP2C9 polymorphism analysis in Han Chinese populations: Building the largest allele frequency database. Pharm. J..

[87] Scordo M.G., Aklillu E., Yasar U., Dahl M.-L., Spina E., Ingelman-Sundberg M. (2001). Genetic polymorphism of cytochrome P450 2C9 in a Caucasian and a black African population. Br. J. Clin. Pharmacol..

