# Supplementary material for: Physiologically Based Pharmacokinetic/Pharmacodynamic Modeling to Predict the Impact of CYP2C9 Genetic Polymorphisms, Co-Medication and Formulation on the Pharmacokinetics and Pharmacodynamics of Flurbiprofen
Source: Pharmaceutics. 2020 Nov 2;12(11):1049. doi: 10.3390/pharmaceutics12111049 (PMC7693160; doi:10.3390/pharmaceutics12111049)
Supplement: Supplementary file 1 [file pharmaceutics-12-01049-s001.pdf]

pharmaceutics

Supplementary Materials: Physiologically Based Pharmacokinetic/Pharmacodynamic Modeling to Predict the Impact of CYP2C9 Genetic Polymorphisms, Co-medication and Formulation on the Pharmacokinetics and Pharmacodynamics of Flurbiprofen

Ioannis Loisios-Konstantinidis, Rodrigo Cristofoletti, Masoud Jamei, David Turner and Jennifer Dressman

pharmaceutics MDPI

Table S1. Summary of main CYP2C9 genotype-based metabolic differences in the default inputs of the Simcyp® North European Caucasian (NEurCaucasian) and Chinese healthy volunteer virtual populations

Parameters	North European Caucasian						Chinese					
	1*/1*	1*/2*	1*/3*	2*/2*	2*/3*	3*/3*	1*/1*	1*/2*	1*/3*	2*/2*	2*/3*	3*/3*
Frequency	0.672	0.186	0.111	0.011	0.017	0.003	0.924	0.0024	0.0712	0	0	0.0024
CYP2C9 abundance in GI tract (pmol/mg)	12.5	11.4	11.4	11.4	11.4	3.4	10.3	9.3	9.3	9.3	9.3	2.8
CYP2C9 abundance in liver (pmol/mg)	83.4	75.8	75.8	75.8	75.8	23	68.5	62.3	62.3	62.3	62.3	18.9
Average liver volume (L)	1.65056						1.402976					

pharmaceutics MDPI

Table S2. Comparison of predicted and observed pharmacodynamic parameters (R_{max}, TR_{max} and AUCE) values of flurbiprofen and calculation of predicted to observed ratio ($R_{pred/obs}$).

PD effect[a]	R_{max} (%)			TR_{max} (h)[b]			AUCE (%·h)			$T_{80\% \ baseline}$ (h)
	obs	pred	$R_{pred/obs}$	obs	pred	$R_{pred/obs}$	obs	pred	$R_{pred/obs}$	Pred
**TPEP amplitude reduction (%)**										
CYP2C9 1*/1*		34.3	0.72		4.00	1.00		187.8	0.84	9.4
CYP2C9 1*/2*	47.3	39.7	0.84	4.00	4.00	1.00	224.2	218.6	0.98	11.9
CYP2C9 1*/3*		45.7	0.97		4.00	1.00		252.6	1.13	16.3
S_{DLM_SI} = 0.125		37.4	0.79		3.48	0.87		196.6	0.88	
S_{DLM_SI} = 0.0054	47.3	36.2	0.77	4.00	3.72	0.93	224.2	205.6	0.92	
S_{DLM_SI} = 0.0712		36.7	0.78		3.60	0.90		202.2	0.90	
S_{DLM_SI} = 0.0018		38.8	0.82		4.44	1.11		201.3	0.90	
**Pain intensity reduction (%)**										
CYP2C9 1*/1*		43.2	0.81		3.36	0.84		224.3	1.00	8.16
CYP2C9 1*/2*	53.1	38.9	0.73	4.00	3.36	0.84	224.2	210.3	0.94	9.12
CYP2C9 1*/3*		42.1	0.79		3.36	0.84		236.3	1.05	12.5
S_{DLM_SI} = 0.125		43.9	0.83		3.36	0.84		202.9	0.90	
S_{DLM_SI} = 0.0054	53.1	45.2	0.85	4.00	3.36	0.84	224.2	204.1	0.91	
S_{DLM_SI} = 0.0712		42.8	0.81		3.12	0.78		206.3	0.92	
S_{DLM_SI} = 0.0018		43.5	0.82		3.84	0.96		203.1	0.91	

[a] Population simulations with healthy Caucasian virtual population. [b] Median value.
